# Digital skull anatomy of the Oligocene North American tortoise *Stylemys nebrascensis* with taxonomic comments on the species and comparisons with extant testudinids of the *Gopherus*–*Manouria* clade

**DOI:** 10.1186/s13358-024-00311-y

**Published:** 2024-03-05

**Authors:** Serjoscha W. Evers, Zahra Al Iawati

**Affiliations:** 1 Department of Geosciences, University of Fribourg, Fribourg, Switzerland; 2 GeoZentrum Nordbayern, Friedrich-Alexander-Universität Erlangen-Nürnberg, Erlangen, Germany

**Keywords:** Tortoises, Testudinidae, Turtles, Taxonomy, Systematics, Cranial evolution, Digital anatomy

## Abstract

The anatomy of North American tortoises is poorly understood, despite a rich fossil record from the Eocene and younger strata. *Stylemys nebrascensis* is a particularly noteworthy turtle in this regard, as hundreds of specimens are known from Oligocene deposits, and as this species is one of the earliest fossil turtles to have been described in the scientific literature. Since its initial description based on a shell, many specimens with more complete material have been referred to *Stylemys nebrascensis*. Here, we review and confirm the referral of an important historic specimen to *Stylemys nebrascensis*, which includes shell, non-shell postcranial, and skull material. This allows us to document unique skull features of *Stylemys nebrascensis* (e.g., an unusual ‘poststapedial canal’ that connects the posterior skull surface with the cavum acustico-jugulare) and to refer another well-preserved skull to the species. Based on computed-tomography scanning of these two skulls, we provide a detailed description of the cranial and mandibular osteology of *Stylemys nebrascensis*. *Stylemys nebrascensis* has a combination of plesiomorphic skull characteristics (e.g., retention of a medial jugal process) and derived traits shared with extant gopher tortoises (e.g., median premaxillary ridge) that suggest it may be a stem-representative of the gopher tortoise lineage. This supports the hypothesis that extant and fossil tortoises from North America form a geographically restricted clade that split from Asian relatives during the Paleogene.

## Introduction

*Stylemys nebrascensis* from the Oligocene of North America is one of the earliest fossil turtles to be recognized and described (Cope, 1884; Hay, 1908; Leidy, 1851). The species is, even historically, known from hundreds of referred specimens (Hay, 1908), but most of these are shells without skull or postcranial associations. The first diagnostic features for *Stylemys nebrascensis* were thus largely based on shell characteristics (Hay, 1908). Nevertheless, Hay (1908) referred two skulls to *Stylemys nebrascensis*, in which he noticed a median premaxillary ridge, a feature otherwise known from North American gopher tortoises (genus *Gopherus*). In addition to other similarities, Hay (1908) interpreted this ridge to indicate a close relationship of *Stylemys nebrascensis* with gopher tortoises, contradicting Cope’s (1884) opinion that *Stylemys nebrascensis* is an emydid turtle, which was never explicitly justified by the author. However, Hay’s (1908) skull referrals were based only on small anatomical overlap in shell materials (e.g., Auffenberg, 1964). Later works by Case (1920, 1925, 1936) established additional non-shell material of *Stylemys nebrascensis* based on more complete specimens that had sufficient overlap with the known shell morphology of the species to rigorously establish these referrals (e.g., Auffenberg, 1964; Vlachos, 2018). This material includes a well-preserved skull (Case, 1925; UMMP 9318). Although the articulation of the lower jaw to the cranium in that specimen made it impossible to assess if the median premaxillary ridge observed by Hay (1908) was also present in the new material, the ridge became accepted as a diagnostic feature (Auffenberg, 1964; Vlachos, 2018), because other aspects of the skull morphology of Case’s specimen matched those described by Hay (1908). Whereas Case considered *Stylemys nebrascensis* once again to be an emydid turtle (Case, 1920, 1925, 1936), all subsequent studies consider the species to be a tortoise (e.g., Auffenberg, 1964; Bramble, 1971; Crumly, 1994; Meylan & Sterrer, 2000; Vlachos, 2018; Vlachos & Rabi, 2018; Williams, 1950, 1952).

Although most studies discuss *Stylemys nebrascensis* in the context of other North American tortoises (e.g., Crumly, 1994), some earlier studies suggested close relationships with taxa from other parts of the world (e.g., Africa: Szalai, 1930) or used the genus “*Stylemys*” for fossil material outside of North America (e.g., “*Stylemys*” *bottii* in Europe: De Stefano, 1902). These hypotheses have largely been dismissed since (e.g., Georgalis et al., 2021; Vlachos, 2018). Nevertheless, the exact phylogenetic position of *Stylemys nebrascensis* among tortoises is contested even among studies using explicit phylogenetical methods (e.g., Vlachos, 2018). The species has variously been interpreted as closely related to gopher tortoises (Crumly, 1994) or alternatively as a stem or crown group Testudininae (i.e., the clade that includes all tortoises but gopher tortoises and *Manouria* spp.; e.g., Meylan & Sterrer, 2000; osteological analysis in Vlachos & Rabi, 2018), sometimes even nested deeply among testudinines as a member of Geochelona (parsimony analysis of molecular and osteological dataset in Vlachos & Rabi, 2018). Some of this phylogenetic uncertainty is expected to derive from the fact that fossil testudinids are comparatively poorly described. This is particularly intriguing for *Stylemys nebrascensis*, given its abundance in the fossil record. For instance, Case’s (1925) work remains the most useful skull description of *Stylemys nebrascensis* to date, but it is short and undetailed based on today’s standards, which were largely established in the landmark work by Gaffney (1979) and superseded only more recently through the use of computed-tomography data (e.g., Brinkman et al., 2006; Gaffney et al., 2006; Jones et al., 2012; Sterli et al., 2010) and the subsequent advent of fully digital skull descriptions based on bone-by-bone segmentations of fossils that also describe internal skull features in some depth (e.g., Evers et al., 2019a; Raselli, 2018). Whereas many shell characters commonly used for phylogenetic studies can readily be scored from superficial shell descriptions and standardized depictions in dorsal and ventral view, many skull characters used for turtle phylogenetics are concealed in standard views of specimens (e.g., Evers & Benson, 2019; Joyce & Bell, 2004). The cranial re-descriptions of fossils are, therefore, a particularly rich source of the novel data needed for achieving more rigorous phylogenetic reconstructions.

Here, we provide a detailed skull description of *Stylemys nebrascensis* as a first step toward a more comprehensive anatomical understanding of fossil North American tortoises. Our work is based on digital dissections from high-resolution micro-computed tomography of two specimens, including the one described by Case (1925) (UMMP 9318).

The hypothesis that *Stylemys nebrascensis* is closely related to gopher tortoises, as well as to other fossil North American tortoises such as Miocene–Pliocene *Hesperotestudo* spp., offers the possibility that North American tortoises form a geographically contained clade (e.g., Bramble, 1971). Recent molecular studies have found North American gopher tortoises as the sister group to Asian testudinids of the genus *Manouria* (e.g., Thomson et al., 2021), with confidence intervals for the divergence times between these groups spanning most of the Eocene and slightly extending into the older parts of the Oligocene (Thomson et al., 2021). If this molecular topology is indeed correct, it is likely that the Oligocene–Pliocene North American fossil tortoises (particularly *Stylemys nebrascensis*, *Oligopherus laticuneus* and *Hesperotestudo* spp.) are on the stem-lineage of gopher tortoises and phylogenetically bracketed by those and *Manouria* spp. For this reason, we also provide segmentations of the skulls of *Gopherus polyphemus* and *Manouria impressa* as comparative taxa within this work.

## Material and methods

We selected two specimens that complement each other in terms of their preservation, so that the complete cranial morphology of *Stylemys nebrascensis* can be described between the two. UMMP 9318 was chosen because it is a historical specimen that was referred to *Stylemys* based on its association with a partial carapace and complete plastron (Case, 1925). We offer some additional verifications of this taxonomic referral. The skull of the specimen is generally well preserved, with the mandible in articulation and only the right squamosal missing. However, as CT scans resolve, the palate region including the snout is badly fractured, obscuring important details of the triturating morphology. The other specimen we used for this study, FMNH UC 1501, is well preserved in the palate, skull roof, and braincase area, but has severely damaged cheek regions. As this specimen is without associated postcranial material, its referral to *Stylemys nebrascensis* is justified in full in the Results section.

The skull of UMMP 9318 was scanned with a Nikon XT H 225ST µCT system housed in the Department of Earth and Environmental Sciences at the University of Michigan. Scans were collected by Adam Rountrey with energy settings of 105 kV and 175 µA and a resolution (isotropic voxel size) of 0.02136 mm. This scan is deposited as an open access file at Deep Blue Data, the file hosting service of the University of Michigan. The CT scan is accessible under this link: 10.7302/74pd-kb09 (CTEES, 2022a). We note that selected postcranial materials of UMMP 9318 (cervical vertebrae, both humeri, right scapula, right coracoid, left femur) were also CT-scanned, although they are not used for this specific study. These postcranial scans can be found at: 10.7302/h3aw-9427 (CTEES, 2022b).

FMNH UC 1501 was scanned with a General Electric phoenix v|tome|x s at the UChicago PaleoCT facility of the University of Chicago. Scans were collected with energy settings of 100 kV and 400 µA and a resolution of 0.0409 mm. This CT scan had previously been made available on the online repository MorphoSource with the Media ID 000357841 (Evers, 2021), and is accessible under this link: https://www.morphosource.org/concern/media/000357841.

Both specimens were segmented manually using the Lasso and interpolation tools of Mimics v. 24–25. For UMMP 9318, we segmented only the right side of the cranium and the left side of the mandible, as these were the better-preserved sides. For FMNH UC 1501, the entire fossil was segmented. The 3D models were deposited as a new MorphoSource project (Evers & Al Iawati, 2023) and are available under this link: https://www.morphosource.org/projects/000516064.

For comparative purposes, we also used CT scans of a specimen each of *Gopherus polyphemus* (FMNH 211815; Evers & Benson, 2018; MorphoSource ID 000042629; link: https://www.morphosource.org/concern/parent/000S10605/media/000042629) and *Manouria impressa* (SMF 69777; Werneburg & Joyce, 2021; MorphoSource ID 000354076; link: https://www.morphosource.org/concern/parent/000354073/media/000354076). The CT scans for these two specimens had previously been uploaded to MorphoSource, and details about the CT scanning procedure and facilities are given with the above links. The 3D models of *Gopherus polyphemus* had also been published previously and are thus part of previously existing MorphoSource projects (Evers & Benson, 2018; http://www.morphosource.org/Detail/ProjectDetail/Show/project_id/462; Evers & Ponstein, 2022; https://www.morphosource.org/projects/000408332). The 3D models of *Manouria impressa* were newly segmented for this project and are thus deposited in the same MorphoSource project in which the *Stylemys nebrascensis* models are kept (i.e., https://www.morphosource.org/projects/000516064). We do not describe these extant species in detail, but provide overview figures and 3D models that document these specimens’ full osteology. In the description below, we often refer to one of these modern species for comparisons. In most cases, we do not repeat the specimen numbers, but all comparisons are based on the above specimens unless otherwise indicated.

For extant taxa, we follow the taxonomy of the Turtle Taxonomy Working Group (TTWG, 2021). For fossil testudinids, the taxonomic revision of Vlachos (2018) provides the baseline for our work. For clade names, we follow the phylogenetic nomenclature as laid out in Joyce et al. (2021). The anatomical nomenclature broadly follows Gaffney (1972) for the cranium, Evers et al. (2022) for the mandible, and Hutchison and Bramble (1981) for plastral scutes.

## Results

### Systematic palaeontology

Testudines Batsch, 1788

Testudinoidea Fitzinger, 1826

Testudinidae Gray, 1825

*Stylemys* Leidy, 1851

*Stylemys nebrascensis* Leidy, 1851

**Fig. 1:**
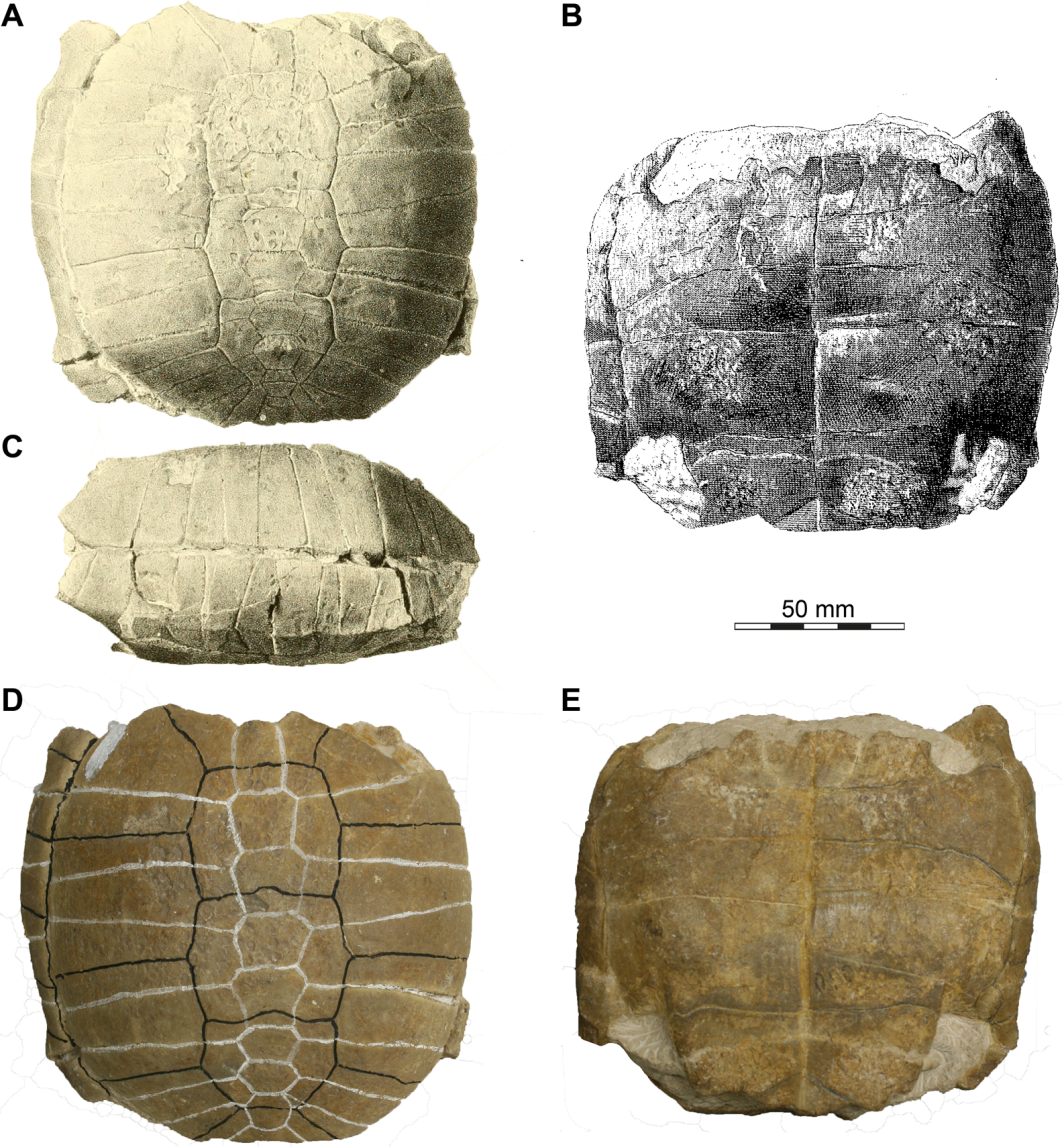
The shell of USNM 97, holotype of *Stylemys nebrascensis*. **A**, **B** Illustrations of the holotype as printed by Leidy (1853: plate IX, figs. 1–2). **C** Illustration of the holotype as printed by Leidy (1952: plate XII A, Fig. 2; note the specimen was originally printed as a mirrored image, which we reverted here). **D**–**E** Recent photographs of the holotype in its current state within the collection of the USNM. **A** dorsal view. **B** ventral view. **C** Left lateral view. **D** Dorsal view. **E** Ventral view. **A**–**C** Are in the public domain, and **D**–**E** were made available to the authors by Evangelos Vlachos

**Fig. 2:**
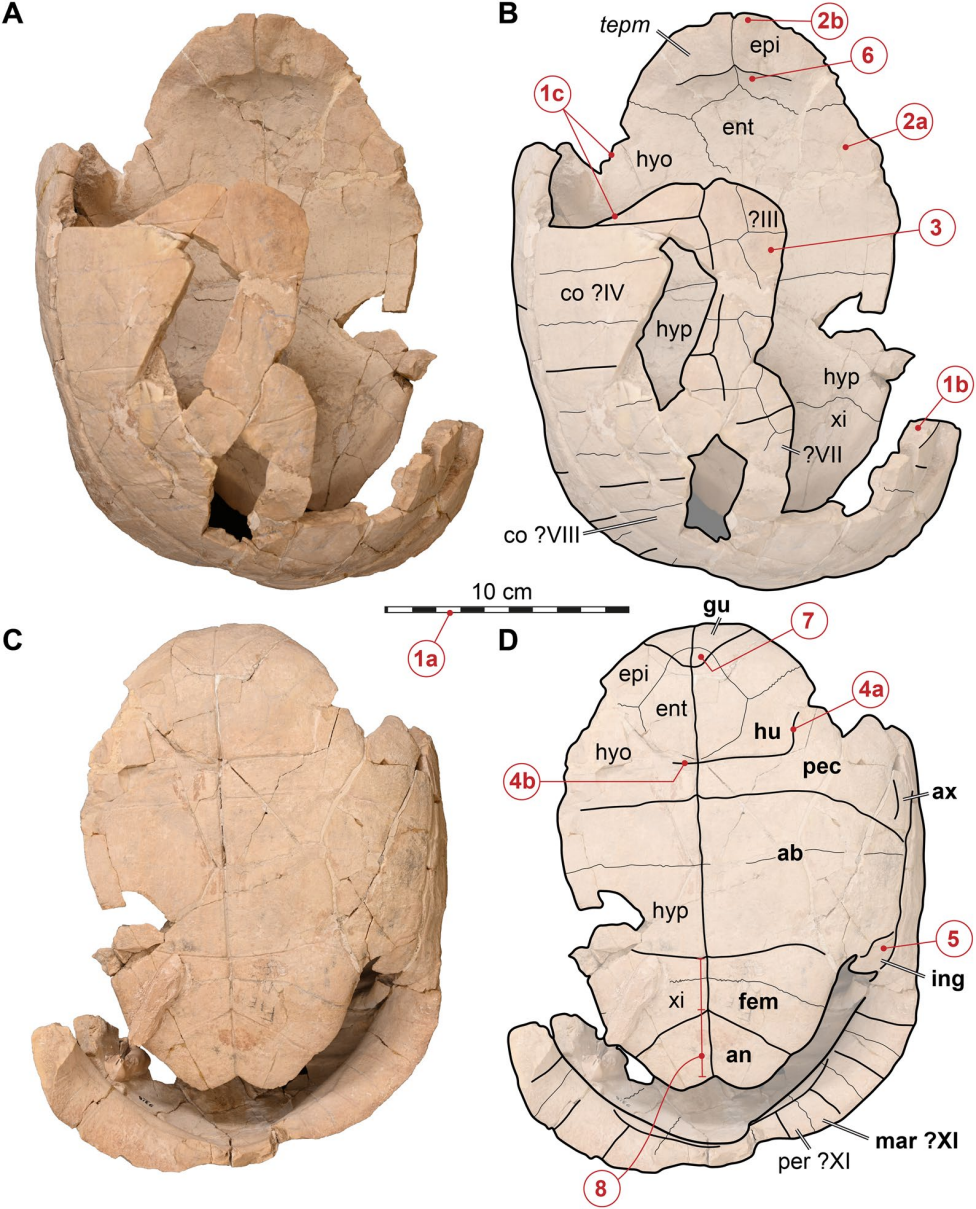
The shell of UMMP 9318, referred to *Stylemys nebrascensis*. **A** Photograph in dorsal view. **B** As A, with interpretative line drawing. **C** Photograph in ventral view. **D** As C, with interpretative line drawing. Note that sutures between shell bones are indicated in a thin line style and labelled in regular font, whereas contacts between scutes are indicated in a thick line style and labelled in bold font. Anatomical features that are not bones or scutes are labelled in italics. Lone Roman numerals indicate neurals. Features labelled in red indicate traits that are important for the taxonomic referral of the specimen to *Stylemys nebrascensis*. Arabic numbers cross-refer to diagnostic traits in full text: 1a, small specimens size; 1b, thick bone; 1c, high shell doming; 2a, broad and short anterior plastral lobe; 2b, absence of gular protrusions; 3, partially preserved neurals with hexagonal shapes with anteriorly short sides; 4a, laterally convex humero-pectoral sulcus; 4b, position of humero-pectoral sulcus posterior to entoplastron; 5, small inguinal scute; 6, shallow fossa posterior to dorsally thickened epiplastral margin; 7, gulars crossing the entoplastron; 8, medially longer anals than femorals. Abbreviations: ab, abdominal; an, anal; ax, axillary; co, costal; ent, entoplastron; epi, epiplastron; fem, femoral; gu, gular; hu, humeral; hyo, hyoplastron; hyp, hypoplastron; ing, inguinal; mar, marginal; pec, pectoral; per, peripheral; tepm, thick epiplastral margin; xi, xiphiplastron. The photographs that form the basis of this figure were taken by Adam Rountrey and are copyrighted by the Regents of the University of Michigan and were shared with the authors under a CC-BY NC 4.0 license

*Type material*. USNM 97 (holotype), a partial shell (Leidy 1852: plate XII A, figs. 1–2; Leidy, 1853: plate XIX: figs. 1–2).

*Remarks*. The holotype of *Stylemys nebrascensis* was not initially figured in the meeting report naming the species (Leidy, 1851). It was first figured by Leidy in 1852 (plate XII A: figs. 1–2; dorsal and ventral view; note the specimen is mirrored) and slightly later again by Leidy (1853: plate XIX: figs. 1–2; dorsal and left lateral view). We are not aware of any additional published illustrations or photos of the specimen. However, photos of the holotype that were made by Evangelos Vlachos are deposited in the online collections of the National Museum of Natural History (EID: http://n2t.net/ark:/65665/34b1ab465-3706-4cd6-96cc-e69ef0afe7a7), and a 3D model is deposited by the institution (link: https://www.si.edu/object/3d/stylemys-nebrascensis-leidy-1851%3Ab1269519-a240-46e5-abb7-29afaae3924f). Here, we provide a composite figure showing historic depictions and various views of the holotype in a single figure (Fig. 1). An interpretation of the sutures and scute sulci have previously been highlighted with colours on the holotype specimen’s carapace by an unknown person, thereby obscuring the original sutural lines seen by Leidy. In his 1852 study, Leidy mentions a supernumerary (nineth) neural bone (‘vertebral’ in Leidy, 1852), which is weakly visible in his drawings (Fig. 1A), but not indicated by the white-coloured sutural interpretations (Fig. 1D).

*Type locality*. Brule Formation (Hutchison, 1996), White River Group, South Dakota, USA. Orellan NALMA (early Rupelian, early Oligocene). See Vlachos (2018) for further details.

*Revised shell-based diagnosis*. *Stylemys nebrascensis* can be differentiated from co-eval and spatially co-occurring tortoises by the following combination of characters: smaller size (< 530 mm straight carapace length) and thick bone structure, especially in the plastron (up to 625 mm carapace length and thinner bones for *Oligopherus laticuneus*; up to 700 mm for “*Testudo*” *brontops*); adult shell high-domed (less domed in *Oligopherus laticuneus* and “*Testudo*” *brontops*); short but transversely broad anterior plastral lobes without strong epiplastral or gular protrusions (elongate anterior plastral lobes with far anteriorly projecting gular protrusions present in *Oligopherus laticuneus* and “*Testudo*” *brontops*); neurals with posteriorly short sides absent, at most neural 2 octagonal (neurals with posteriorly short sides or several octagonal neurals present in *Oligopherus laticuneus* and “*Testudo*” *brontops*); humero-pectoral sulcus positioned posterior to entoplastron (crossing entoplastron in “*Testudo*” *brontops*) and laterally strongly curved toward the anterior (not curved in *Oligopherus laticuneus*); inguinal scale small (large in “*Testudo*” *brontops*) and never contacting the femoral scale.

*Revised cranial diagnosis*. *Stylemys nebrascensis* can be differentiated from the co-eval and spatially co-occurring *Oligopherus laticuneus* based on well-developed jugal contacts with the palatine and pterygoid (absent in *Oligopherus laticuneus*); an anteroposteriorly more elongated, triangular parabasisphenoid (roughly pentagonal, as long as wide in *Oligopherus laticuneus*); a weak anterior constriction of the interorbital fenestra (strong constriction caused by ventrally deep crista cranii and dorsally raised vomer in *Oligopherus laticuneus*); a small postorbital–squamosal contact (broad in *Oligopherus laticuneus*); a robust but low interpremaxillary ridge (deep and thin in *Oligopherus laticuneus*).

In the discussion, we additionally dedicate a section to discussing potential other diagnostic or otherwise noteworthy skull features of *Stylemys nebrascensis* (see below).

### Referral of both specimens to *Stylemys nebrascensis*

**Fig. 3:**
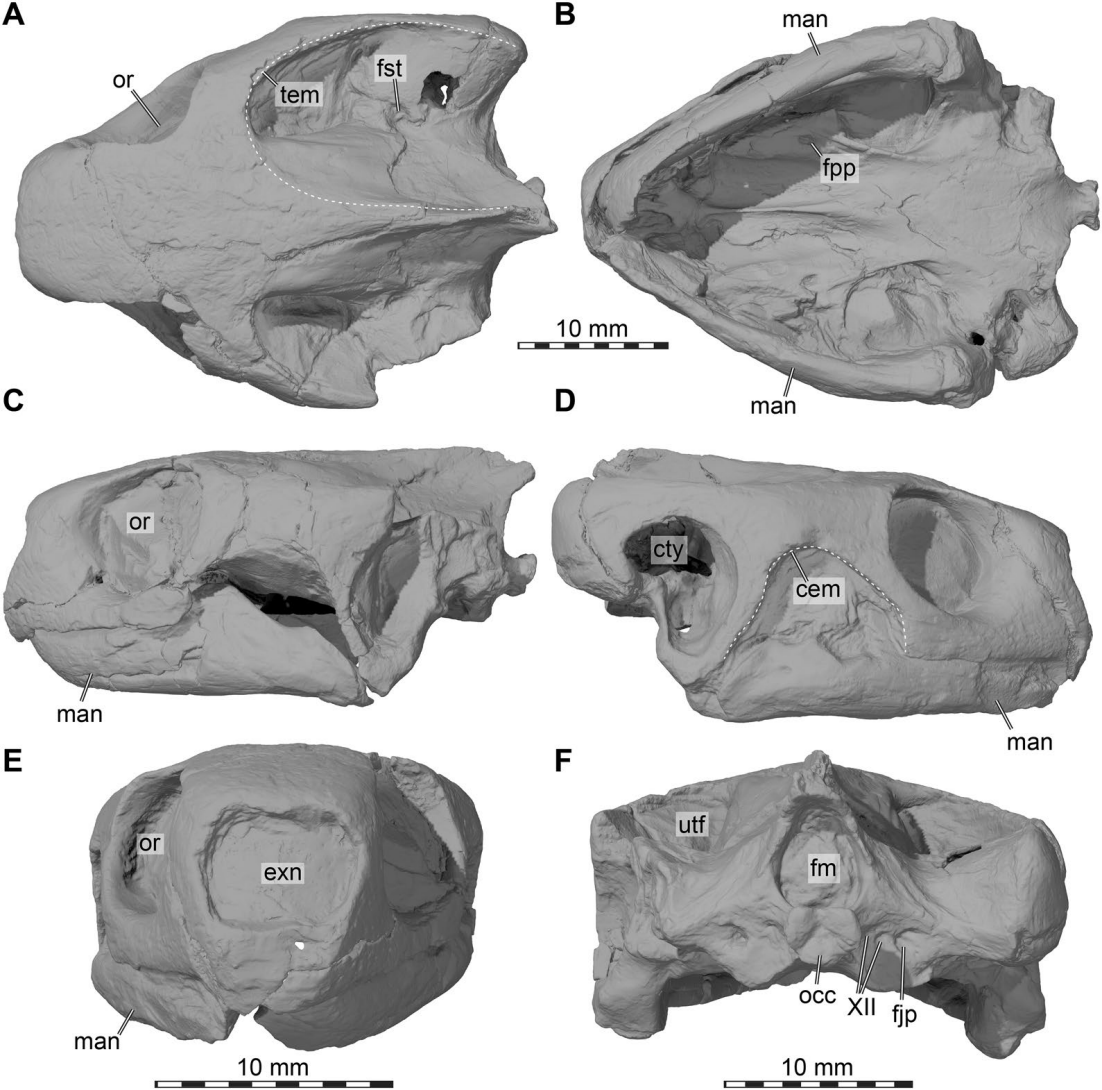
3D renderings of the cranium of UMMP 9319, referred to *Stylemys nebrascensis*, as preserved in the fossil. **A** Dorsal view. **B** Ventral view. **C** Left lateral view. **D** Right lateral view. **E** Anterior view. **F** Posterior view. Note that the mandible is articulated to the cranium. cem, cheek emargination; cty, cavum tympani; exn, external naris; fpp, foramen palatinum posterius; fjp foramen jugulare posterius; fst, foramen stapedio-temporale; man, mandible; occ, occipital condyle; or, orbit; tem, upper temporal emargination; XII, hypoglossal nerve (CN XII) foramina

Case (1925) referred UMMP 9318 to *Stylemys nebrascensis*. The specimen includes a partly complete carapace and complete plastron (Fig. 2) alongside postcranial elements and the nearly complete skull (Fig. 3). The specimen comes from an “Oligocene clay” locality along the Hat Creek in Niobrara County, Wyoming (Case, 1925), which belongs to the Brule Formation (Orellan North American Land Mammal Age) of the White River Group (Hutchison, 1996). Therefore, the specimen is from the same formation as the holotype of *Stylemys nebrascensis*. Its taxonomic referral to *Stylemys nebrascensis* has not been questioned in subsequent work (e.g., Auffenberg, 1964; Hutchison, 1996; Vlachos, 2018), although Case (1925) made no specific justification for his referral and although the shell was never figured. We herein figure the shell of UMMP 9318 (Fig. 2) and discuss eight features of the shell that, taken together, confirm the referral of the specimen to *Stylemys nebrascensis* under the current taxonomic framework established by Vlachos (2018) and our revised diagnosis (see above). Specifically, we note that UMMP 9318 is highly domed, only approximately 25 cm long, and has thick bones. The anterior plastral lobe is broad and short and lacks gular protrusions. Although the first two neurals are missing, the remaining neural column lacks neurals with short posterior sides or octagonal neurals. Though incomplete, the humero-pectoral sulcus appears to be medially straight, anterolaterally convex, and located posterior to the entoplastron. The inguinal scute is small and does not contact the femoral. In all regards, this overlaps with the diagnosis of *Stylemys nebrascensis* outlined above. Finally, the epiplastron lacks a dorsal excavation. Whereas many testudinoids show a dorsally thickened epiplastral margin along the anterior plastral lobe, this margin is relatively deeply excavated by a fossa in *Gopherus* spp. and *Oligopherus laticuneus* according to Auffenberg (1964). However, Hutchison (1996) attests *Oligopherus laticuneus* a relatively shallow excavation. Overall, this feature is quite gradational in testudinoids (Joyce & Bell, 2004), such that its taxonomic value among testudinids from the White River Group needs to be more thoroughly assessed in future work. For now, we note that UMMP 9318 has a shallow fossa (Fig. 2A, B) and that this could potentially support a referral to *Stylemys* spp.

The aforementioned combination of traits allows the referral of UMMP 9318 to *Stylemys nebrascensis* and precludes alternative identifications (i.e. *Oligopherus laticuneus*, “*Testudo*” *brontops*, *Hesperotestudo* sp.). UMMP 9318 also lacks features that are listed as diagnostic for the other two valid *Stylemys* species by Vlachos (2018). Although these species have either no geographic (*Stylemys capax*) or temporal (*Stylemys inusitata*) overlap with *Stylemys nebrascensis*, we list these morphological features here. Specifically, UMMP 9318 has gulars that are crossing the entoplastron (feature 7 in Fig. 2D), whereas *Stylemys inusitata* has short gulars not extending onto the entoplastron (Vlachos, 2018; see Hay, 1908: plate 68). We can also rule out the possibility that UMMP 9318 belongs to *Stylemys capax*, because UMMP 9318 has anal scutes that are longer in their midline contact than the femorals (feature 8 in Fig. 2D), whereas the reverse condition is diagnostic for *Stylemys capax* according to Vlachos (2018; Hay, 1908: Fig. 499; this seems to be primarily caused by a deep posterior inter-xiphiplastral embayment that shortens the midline contact of the anals on the posterior plastral lobe).

**Fig. 4:**
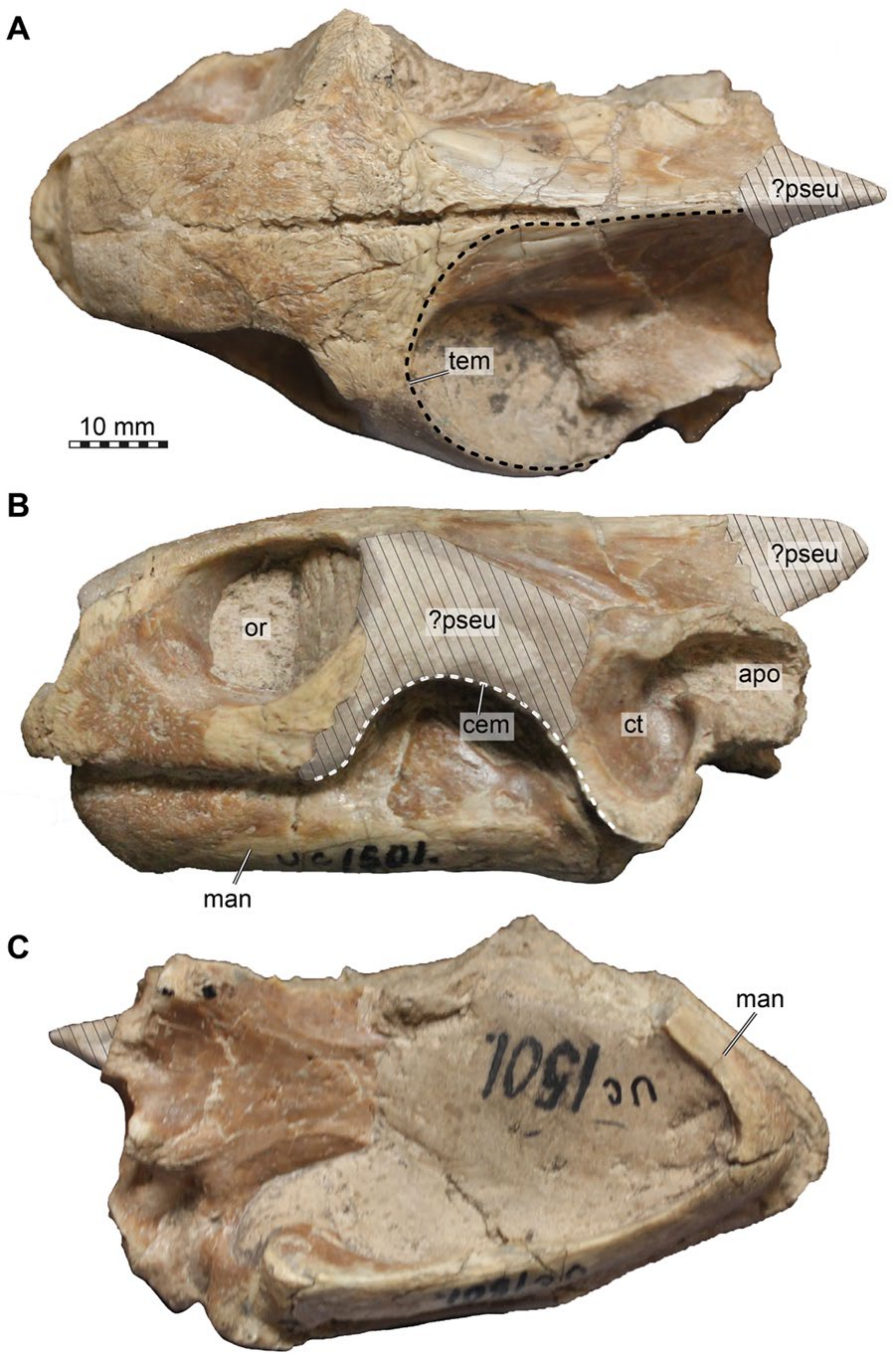
Photographs of the fossil FMNH UC 1501, referred to *Stylemys nebrascensis*. **A** Dorsal view. **B** Left lateral view. **C** Ventral view. Note that sediment covers large parts of the palate, that the mandible is articulated to the cranium, and that some sediment areas are approximate infills of original morphology (left cheek, supraoccipital crest), and it is not entirely clear if these were modelled using a mixture of plaster and sediment, or if they represent sediment pseudomorphs of the original morphology. Abbreviations: ?pseu, possible sediment pseudomorphs; apo, antrum postoticum; cem, cheek emargination; ct, cavum tympani; man, mandible; or, orbit; tem, temporal emargination

FMNH UC 1501 is an isolated skull (Fig. 4). As the holotype of *Stylemys nebrascensis* consists of shell material, the referral of FMNH UC 1501 to the species needs to be justified. Although the specimen has not been described before, Crumly (1984) already lists it taxonomically as *Stylemys nebrascensis*. The museum label further says “Oligocene, Sioux Co., Nebraska, Cedar Creek” (and we could not find additional information on the locality), which is consistent with the stratigraphic and geographic range of *Stylemys nebrascensis* (Vlachos, 2018), and geographically close to both the locality of UMMP 9318 and the type locality. However, the strongest evidence to refer the specimen to *Stylemys nebrascensis* comes from morphological comparisons with UMMP 9318, which can be shown to belong to the species (see above), as already argued by Case (1925).

The skull of FMNH UC 1501 shares important features with that of UMMP 9318, including many of the newly proposed diagnostic features (above), justifying its referral to *Stylemys nebrascensis*. Particularly indicative are features absent in *Oligopherus laticuneus*, such as the presence of a medial process of the jugal with resulting jugal contacts with the palatine and pterygoid, the anteriorly elongate, triangular shape of the parabasisphenoid in ventral view, and the large and unconstricted interorbital fenestra. FMNH UC 1501 also shares with UMMP 9318 feature that are generally unusual for testudinids, but which could so far not be further evaluated for *Oligopherus laticuneus*. Among those are the shape of the processus trochlearis oticum, the presence of a subcavernosal foramen and canal between quadrate and pterygoid, the complete pterygoid enclosure of the foramen posterius canalis carotici interni (fpcci), the position of the anterior foramen for the vidian nerve on the lateral surface of the pterygoid slightly anterior to the epipterygoid position, and the broad, large dorsal foramen into the fossa Meckelii in the mandible. Most of these features cannot be seen in the fossil as it is preserved (Fig. 4) due to the articulation of the mandible and cranium and the sediment cover of the palate and internal skull surfaces, thus highlighting once again the importance of CT scanning for palaeontological applications.

### Description

**Fig. 5:**
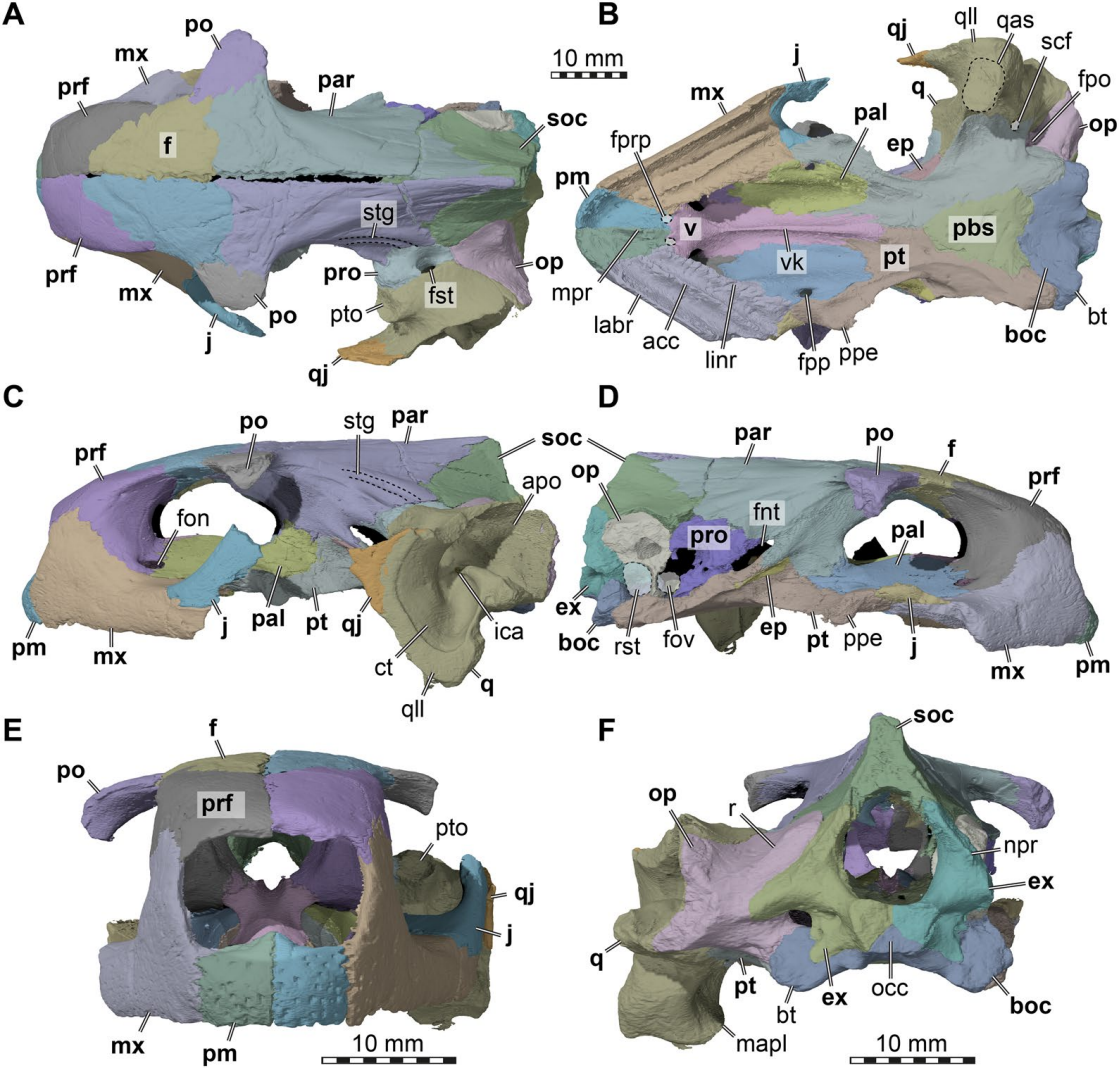
3D renderings of the cranium of FMNH UC 1501, referred to *Stylemys nebrascensis.* **A** Dorsal view. **B** Ventral view. **C** Left lateral view. **D** Right lateral view. **E** Anterior view. **F** Posterior view. Note that bones are labelled in bold font, and traits in regular font. acc, accessory ridge; apo, antrum postoticum; boc, basioccipital; bt, basioccipital tubercle; ct, cavum tympani; ep, epipterygoid; ex, exoccipital; f, frontal; fon, foramen orbito-nasale; fov, fenestra ovalis; fnt, foramen nervi trigemini; fpcci, foramen posterius canalis carotici interni; fpo, fenestra postotica; fpp, foramen palatinum posterius; fprp, foramen praepalatinum; fst, foramen stapedio-temporale; ica, incisura columellae auris; j, jugal; labr, labial ridge; linr, lingual ridge; mapl, medial articular process lip; mpr, medial premaxillary ridge; mx, maxilla; occ, occipital condyle; op, opisthotic; par, parietal; pal, palatine; pbs, parabasisphenoid; pm, premaxilla; po, postorbital; ppe, processus pterygoideus externus; prf, prefrontal; pro, prootic; pt, pterygoid; pto, processus trochlearis oticum; q, quadrate; qas, quadrate articulation surface; qll, quadrate lateral lamina; qj, quadratojugal; soc, supraoccipital; stg, stapedial groove; rst, recessus scalae tympani; v, vomer; vk, vomer keel

**Fig. 6:**
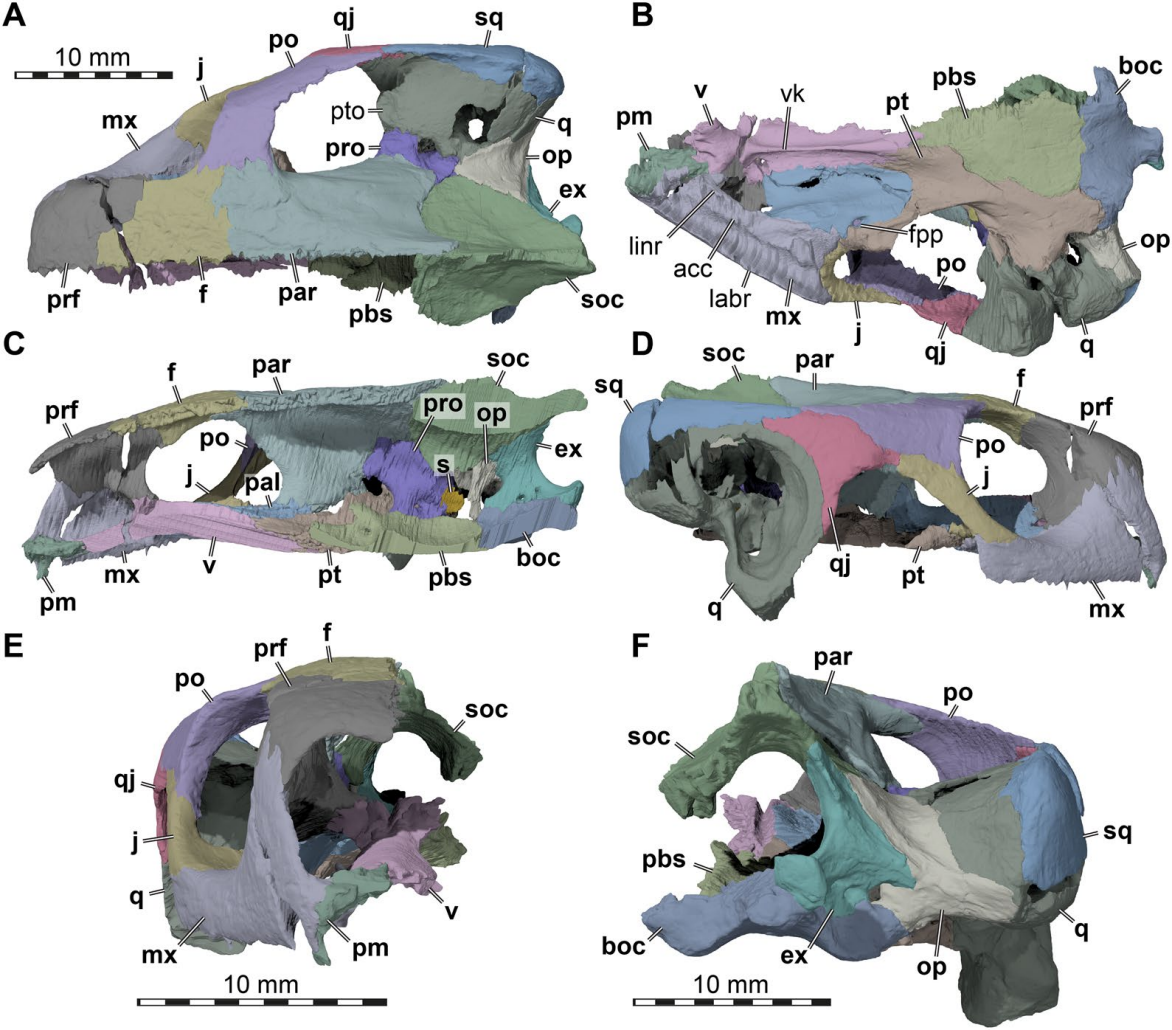
3D renderings of the right side of the cranium of UMMP 9318, referred to *Stylemys nebrascensis*. **A** Dorsal view. **B** Ventral view. **C** Medial view onto right cranial side. **D** Right lateral view. **E** Anterior view. **F** Posterior view. Note that bones are labelled in bold font, and traits in regular font. acc, accessory ridge; boc, basioccipital; ep, epipterygoid; ex, exoccipital; f, frontal; fpp, foramen palatinum posterius; j, jugal; labr, labial ridge; linr, lingual ridge; mx, maxilla; op, opisthotic; par, parietal; pal, palatine; pbs, parabasisphenoid; pm, premaxilla; po, postorbital; prf, prefrontal; pro, prootic; pt, pterygoid; pto, processus trochlearis oticum; q, quadrate; qj, quadratojugal; s, stapes; soc, supraoccipital; sq, squamosal; v, vomer; vk, vomer keel

In the below description of FMNH UC 1501 (Fig. 5) and UMMP 9318 (Fig. 6), the preservational state for each of the two *Stylemys nebrascensis* specimens is mentioned, before the morphology of the species is described. Unless otherwise stated, both specimens document the described morphology.

### Prefrontal

In FMNH UC 1501, both prefrontals are preserved (Fig. 5). While the right prefrontal is entirely complete, the left prefrontal only seems to preserve the posterior of the bone. Anteriorly, a large part of the bone is replaced by a material that has a darker contrast in the CT images, but which seems to preserve the shape of the missing piece. It seems possible that the dark material represents a sediment infill, which replaces the bone in this part of the skull but preserved its morphology. In UMMP 9318, both prefrontals are preserved but the segmented right one is affected by a large fracture (Figs. 3, 6).

The prefrontal of *Stylemys nebrascensis* is a bone in the anterior part of the skull, and involved in the formation of the external naris and the orbit (Figs. 5, 6). It contacts its counterpart across the skull midline, forming the dorsal margin of the external naris. Posteriorly, the prefrontal contacts the frontal in the skull roof. The prefrontal additionally contacts the maxilla, palatine and vomer along its ventral process (Figs. 5, 6).

The prefrontal is broadly exposed in the skull roof, and the contact with the frontal extends from the orbit anteromedially to the skull midline. As a result, the contact between the frontals and prefrontals appears as a “V”-shape in dorsal view (Figs. 5A, 6A). The posterolateral part of the prefrontal extends up to roughly the mid-length of the orbit. As such, the prefrontal forms the largest contribution to the orbit of all bones involved in this structure. Anteriorly, the prefrontals form the dorsal margin of the external naris.

The ventral process of the prefrontal of *Stylemys nebrascensis* is largely buttressed by the maxilla, and specifically by its ascending process (Figs. 5C, D, 6D). Posteriorly, the prefrontal contacts the palatine in the floor of the orbital fossa. Together with this bone, it forms the foramen orbito-nasale, to which neither the maxilla nor the vomer contributes (Figs. 5C, D, 6D). The same condition is found in *Manouria impressa* and *Gopherus polyphemus*. In *Stylemys nebrascensis*, the prefrontal contacts the vomer ventromedially, and this contact bridges over the narial passage. In this prefrontal–vomer contact area, *Gopherus* spp. is reported to have a peculiar small fossa, the so-called prefrontal pit of Crumly (1994), which we can confirm to be present in our specimen of *Gopherus polyphemus*. *Stylemys nebrascensis* and *Manouria impressa* lack this pit.

### Frontal

**Fig. 7:**
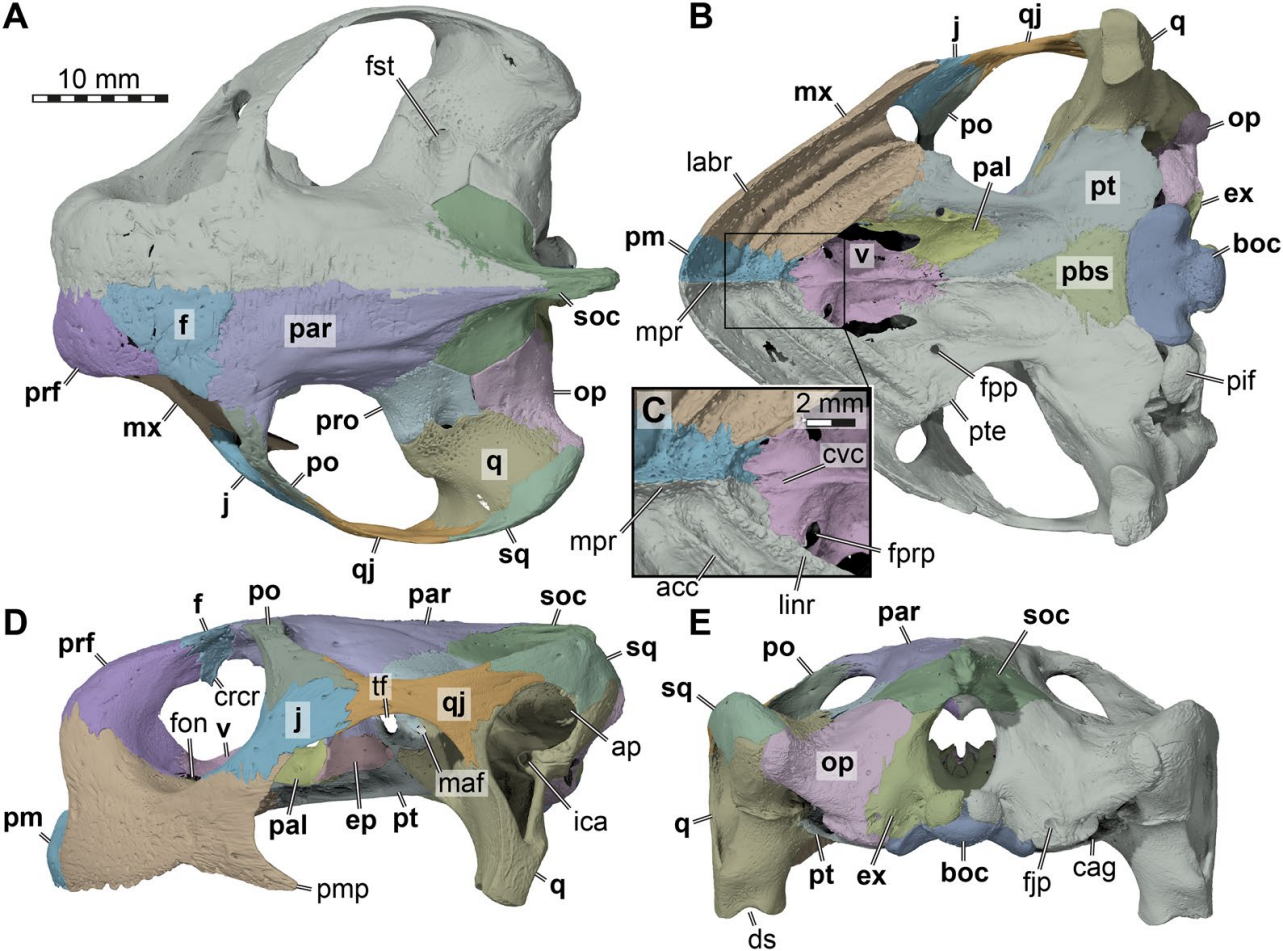
3D renderings of the cranium of *Gopherus polyphemus* (FMNH 211815), with the left cranial side segmented bone by bone. **A** Dorsal view. **B** Ventral view. **C** Close-up of premaxillary-vomerine area. **D** Left lateral view. **E** Posterior view. Note that bones are labelled in bold font, and traits in regular font. acc, accessory ridge; ap, antrum postoticum; boc, basioccipital; cag, carotid artery groove; crcr, crista cranii; cvc, central vomerine cleft; ds, deeply embayed sulcus of articular process, ep, epipterygoid; ex, exoccipital; f, frontal; fjp, foramen jugulare posterius; fon, foramen orbito-nasale; fpp, foramen palatinum posterius; fprp, foramen praepalatinum; fst, foramen stapedio-temporale; ica, incisura columellae auris; j, jugal; labr, labial ridge; linr, lingual ridge; maf, mandibular artery foramen; mpr, median premaxillary ridge; mx, maxilla; op, opisthotic; par, parietal; pal, palatine; pbs, parabasisphenoid; pif, processus interfenestralis; pm, premaxilla; pmp, posterior maxillary process; po, postorbital; prf, prefrontal; pro, prootic; pt, pterygoid; pte, processus pterygoideus externus; q, quadrate; qj, quadratojugal; soc, supraoccipital; sq, squamosal; tf, trigeminal foramen; v, vomer

**Fig. 8:**
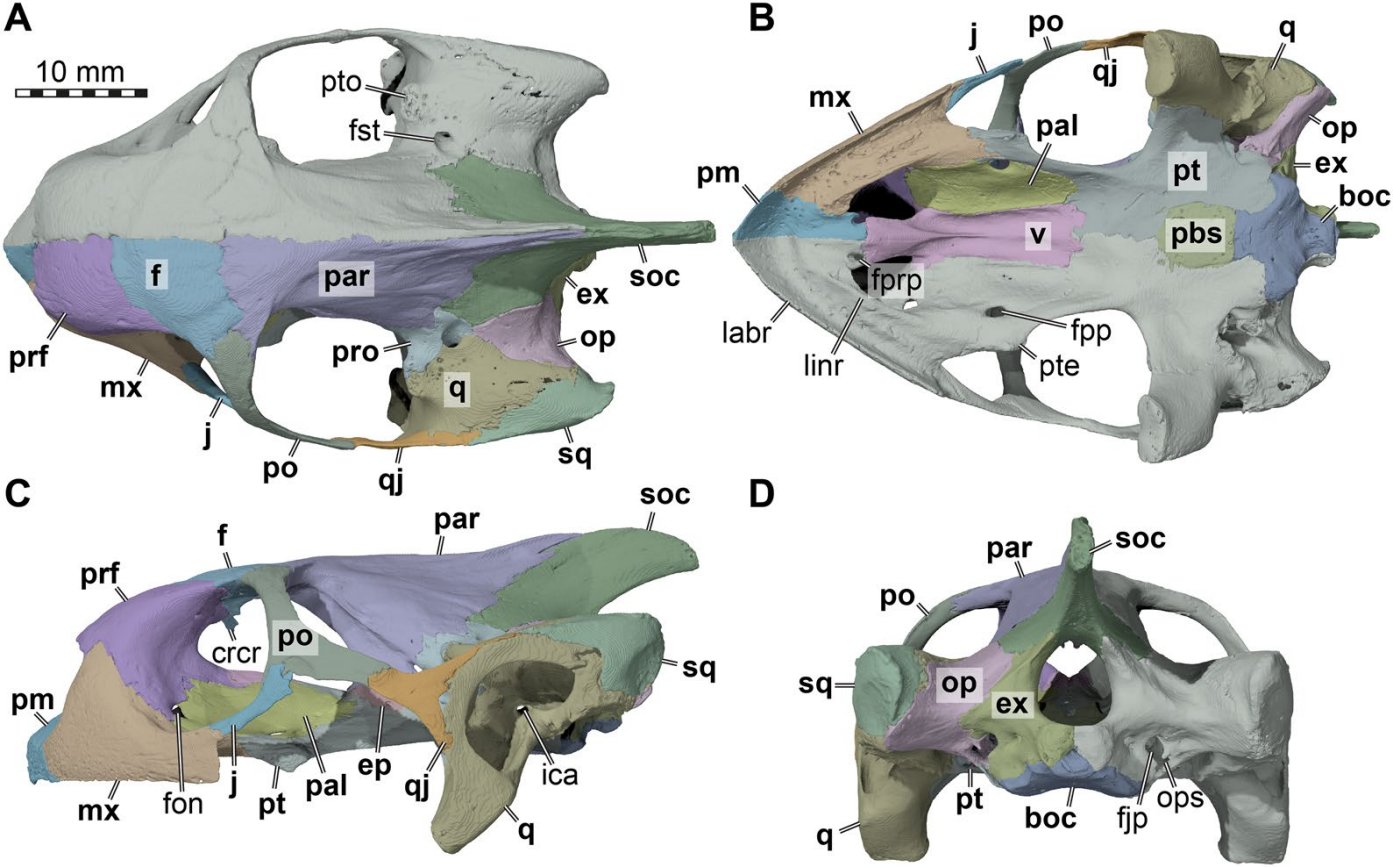
3D renderings of the cranium of *Manouria impressa* (SMT 69777), with the left cranial side segmented bone by bone. **A** Dorsal view. **B** Ventral view. **C** Left lateral view. **D** Posterior view. Note that bones are labelled in bold font, and traits in regular font. boc, basioccipital; crcr, crista cranii; ep, epipterygoid; ex, exoccipital; f, frontal; fjp, foramen jugulare posterius; fon, foramen orbito-nasale; fpp, foramen palatinum posterius; fprp, foramen praepalatinum; fst, foramen stapedio-temporale; ica, incisura columellae auris; j, jugal; labr, labial ridge; linr, lingual ridge; mx, maxilla; op, opisthotic; ops, oropharyngeal slit, par, parietal; pal, palatine; pbs, parabasisphenoid; pm, premaxilla; po, postorbital; prf, prefrontal; pro, prootic; pt, pterygoid; pte, processus pterygoideus externus; pto, processus trochlearis oticum; q, quadrate; qj, quadratojugal; soc, supraoccipital; sq, squamosal; v, vomer

In both specimens of *Stylemys nebrascensis* used herein, both frontals are fully preserved (Figs. 5, 6). The frontal contacts the prefrontal anteromedially, the parietal posteriorly, and the postorbital posterolaterally (Figs. 5A, C, D, E, 6A, C, D, E). The frontals contact one another along their midline and narrow anteriorly into a V-shaped projection. This can also be noticed in *Gopherus polyphemus* (Fig. 7A), whereas the conjunction is rather U-shaped due its low angle contact with the prefrontal in *Manouria impressa* (Fig. 8A).

The lateral margin of the frontal of *Stylemys nebrascensis* contributes to the formation of the orbit, but it is less dominant than the prefrontal and postorbital (Figs. 5A; 6A, D). Along the orbital margin, the frontal contacts the postorbital. This contact is only variably present in testudinids, as it is present in *Manouria impressa* (Fig. 8A, C), but absent in *Gopherus polyphemus* (Fig. 7A, D).

On the ventral surface of the frontal of *Stylemys nebrascensis* there is a distinct ridge, the crista cranii, that extends along the entire length of the bone but becomes shallower posteriorly. Moreover, the crista cranii defines the sulcus olfactorius, which is a ventrally open median trough where the olfactory nerves pass through to the nasal capsule. Anteriorly, the crista cranii gets ventrally deeper, and forms a short medially projecting process together with the prefrontal. This process defines the fissure ethmoidalis, as is also the case in *Gopherus polyphemus*. In some testudinids, including *Manouria impressa*, the right and left processes of the crista cranii meet in the skull midline, thereby forming a circular passage of the fissure ethmoidalis that is fully separated from the ventrally located sulcus vomeri.

### Parietal

**Fig. 9:**
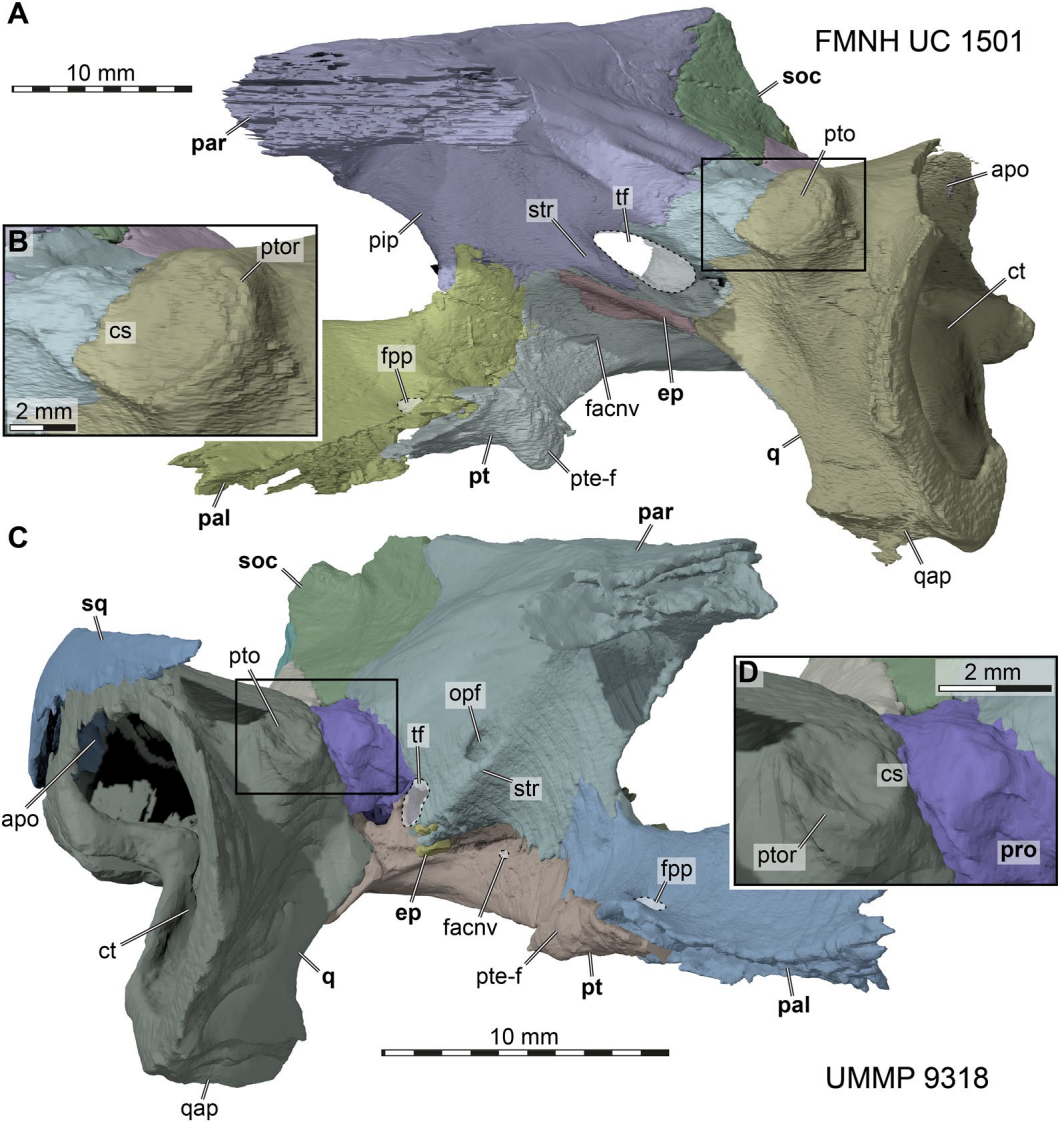
3D renderings of the trigeminal area of *Stylemys nebrascensis*. **A **Left partial cranium of FMNH UC 1501 in anterolateral view. **B** Close-up of processus trochlearis oticum. **C** Right partial cranium of UMMP 9318 in anterolateral view. **D** close-up of processus trochlearis oticum. Note that bones are labelled in bold font, and traits in regular font. apo, antrum postoticum; cs, concave surface of processus trochlearis oticum; ct, cavum tympani; ep, epipterygoid; facnv, foramen anterius canalis nervi vidiani; fpp, foramen palatinum posterius; opf, ophthalmic foramen; pal, palatine; par, parietal; pip, processus inferior parietalis; pro, prootic; pt, pterygoid; pte-f, flange of processus pterygoideus externus; pto, processus trochlearis oticum; ptor, processus trochlearis oticum ridge; q, quadrate; qap, quadrate articular process; soc, supraoccipital; str, subtrigeminal ridge; sq, squamosal; tf, trigeminal foramen

Both parietals are fully preserved in both specimens (Figs. 3, 5 and 6), but the morphology of the ventral part of the descending process is better preserved in FMNH UC 1501, due to breakage in the trigeminal area of UMMP 9318 (Fig. 9). On the skull roof, the parietal of *Stylemys nebrascensis* has a contact with the frontal anteriorly, the postorbital anterolaterally and its counterpart medially (Figs. 5A, 6A). The posterior process overlies the supraoccipital and the prootic posteroventrolaterally. The descending process of the parietal contacts the pterygoid and epipterygoid posteriorly, and the palatine anteriorly (Fig. 9).

The parietal surface that is exposed in the upper temporal fossa of FMNH UC 1501 is marked by a curved, subtle trough, which extends anterodorsally away from the foramen stapedio-temporale (Figs. 5C, 9A). We thus believe that the trough indicated the anterior course of the stapedial artery or one of its subsidiary arteries. The trough is not evident in the smaller specimen UMMP 9318 (Fig. 9C), and we think it is possible that this represents ontogenetic variation.

Along the descending process, the parietal forms the dorsal margin of the trigeminal foramen, which is oval and obliquely oriented in *Stylemys nebrascensis* (Figs. 5C, D, 6C, 9). The parietal extends around the foramen to contact both the prootic posteriorly and the pterygoid ventrally, which are the other bones that contribute to the trigeminal foramen (Fig. 9). The contact with the pterygoid is laterally thickened to a subtrigeminal ridge, which forms a laterally overhanging surface to which’s ventral side the epipterygoid articulates (Fig. 9). Anterodorsally to the trigeminal foramen, the parietal of UMMP 9318 bears a large foramen that is associated to a canal which directs posteroventrally through the descending process, and opens on the medial surface of the braincase just anterior to the cavum epiptericum (Fig. 9C). We hypothesize that this canal is associated to the ophthalmic branch of the trigeminal nerve, and thus call the canal and external foramen ophthalmic canal and ophthalmic foramen, respectively. Ophthalmic foramina are present in some extant tortoises, such as *Testudo marginata* (FMNH 51672) or *Chelonoidis* sp. (SMF 67582), but they are absent in gopher tortoises or *Manouria impressa*. FMNH UC 1501 also lacks the foramen (Fig. 9A), indicating that there can be intraspecific variation to the presence of the canal.

### Postorbital

The postorbitals are incomplete on both sides in FMNH UC 1501, but are completely preserved in UMMP 9318 (Figs. 3, 5A, C, D, E, 6A, D, E). The postorbital of *Stylemys nebrascensis* contacts the frontal anterolaterally and the parietal posterolaterally in the skull roof, the jugal anteroventrally in the orbital margin, and the quadratojugal posteroventrally in the cheek. A point contact with the squamosal is additionally realized dorsal to the quadratojugal (Fig. 6A, D).

The postorbital of *Stylemys nebrascensis* is triradiate, and has a narrow medial process that arches from the vertically oriented cheek region to the horizontal plate of the skull roof (Fig. 6E). This medial process forms a relatively narrow bony bar between the orbit anteriorly and the upper temporal fossa posteriorly (Figs. 5A, 6A). The anterior contact of this process with the frontal is present in most turtles, including *Manouria impressa* (Fig. 8A), *Gopherus agassizii*, and *Gopherus flavomarginatus*. It is, however, absent in our sampled specimen of *Gopherus polyphemus* (Fig. 7A). The postorbital of *Stylemys nebrascensis* contributes to the strong upper temporal emargination (Figs. 3A, 5A, 6A), which exposed the full otic process. The anteroventral process of the postorbital is short, as the jugal remains broadly exposed in the orbit (Fig. 6D, E). The largest process of the postorbital is the posterior one, which is plate-like but tapers toward the squamosal contact (Fig. 6D).

### Jugal

In FMNH UC 1501, the jugal is nearly completely preserved on the left side, with only dorsal portions near the postorbital and quadratojugal contacts missing (Fig. 5A, B, C, E). On the right side, only a small chunk of the jugal is preserved (Fig. 5D). In UMMP 9318, both jugals are preserved, but the left one is fragmented due to a large break in this area of the fossil (Figs. 3C, D, 6A, B, D, E). The jugal of *Stylemys nebrascensis* is laterally limited to a narrow bar between the orbit and the deep cheek emargination (Figs. 5C, 3C, D; 6D). Anteriorly, it is in contact with the maxilla anteriorly and ventrally, and with the postorbital and quadratojugal along its posterior part. The jugal abuts on top of the maxilla, and does not extend ventrally to the level of the labial skull margin (Figs. 5C, 6D).

Additionally, there are contacts with the palatine and the pterygoid posteromedially along a medial process that extends from the orbital fossa (Figs. 5B; 6B, C). This medial process of the jugal is generally present in turtles, but absent in species of the *Gopherus*–*Manouria* clade (Figs. 7, 8), as well as other testudinids (e.g., *Testudo marginata*), which thus lack a jugal–pterygoid or jugal–palatine contact.

### Quadratojugal

The quadratojugal is only poorly preserved on the left side of FMNH UC 1501 (Fig. 5A–C), where it seems to be replaced by a sediment pseudomorph which preserved the rough outline of the bone, including the cheek emargination (Fig. 4C). It is not entirely clear if this area was modelled with a mixture of plaster and sediment. However, the quadratojugal is completely preserved on both sides of UMMP 9318 (Figs. 3C, D, 6D), providing the genuine morphology of the element. As in other testudinids, the quadratojugal of *Stylemys nebrascensis* is a thin triradiate bone, which contributes to the formation of the posterodorsal margin of the deep cheek emargination (Figs. 3D, 6D). The posteroventral process of the quadratojugal extends along the anterior margin of the quadrate, but only until about half the height of the cavum tympani, so that it does not reach the mandibular condyle (Figs. 5C, 6D). The quadratojugal does not contribute to the cavum tympani, as it articulates with its posterior margin to the medial surface of the quadrate (Figs. 5C, 6D). The short posterodorsal process of the quadratojugal is in contact with the squamosal (Figs. 6A, D), as in *Gopherus polyphemus* and *Manouria impressa* (Figs. 7, 8). Anterodorsally, the quadratojugal of *Stylemys nebrascensis* has a long contact with the postorbital. The anterior process is broken in FMNH UC 1501 but complete in UMMP 9318, in which it reaches the jugal (Fig. 6D), as in *Gopherus polyphemus* (Fig. 7D). In *Manouria impressa*, the jugal is only in contact with the postorbital, but not with the jugal (Fig. 8C).

### Squamosal

Both squamosals are not preserved in FMNH UC 1501 (Fig. 5). In UMMP 9318, the left squamosal is absent, but the right one is complete and articulated (Figs. 3A; 6A, D, F; 9C). The squamosal contacts the quadrate, the quadratojugal, and the postorbital (Fig. 6A, D). The bone has a relatively simple morphology, without posterodorsal crests or processes (Fig. 6D). Instead, the posterior part of the squamosal is formed as a simple, posterodorsally convex cover over the large antrum postoticum (Fig. 6D). This posterodorsal extension of the cavum tympani is fully circumvented by the quadrate, and the squamosal caps the respective opening, deepening the antrum with its anteriorly concave surface (Fig. 6D). The squamosal is, however, excluded from contributing to the margin of the cavum tympani (Fig. 6D). Anteriorly, the squamosal forms a thinning process, which contacts the quadratojugal and postorbital (Fig. 6A, D). Medially, the squamosal reaches only shortly into the dorsal surface of the upper temporal fossa, so that the quadrate remains broadly exposed in dorsal view (Fig. 6A).

### Premaxilla

The premaxillae of UMMP 9318 are badly preserved, and broken particularly along their ventrally facing triturating surfaces (Figs. 3E, 6B, E). This is insofar unlucky, in that the specimen thus cannot provide information about the presence or absence of a median interpremaxillary ridge, which was proposed as an early recognized, cranial diagnostic feature of *Stylemys nebrascensis*. However, the premaxillae are perfectly preserved in FMNH UC 1501 (Fig. 5B, E). The premaxilla forms the anterior tip of the snout, and an interpremaxillary ridge is clearly present (see below; Fig. 5B). The premaxilla of *Stylemys nebrascensis* contacts the maxilla laterally, the vomer posteriorly and is conjoined by its counterpart at the midline of the skull. (Fig. 5B, E).

The premaxilla forms the ventral margin of the external naris. Along this margin, and at the skull midline where right and left premaxillae meet, there is a dorsally projecting spike-like process, which shallowly divides the external naris into left and right valves (Fig. 5E). This process is at the anterior margin of the external naris, as is also the case in G*opherus polyphemus*. In *Manouria impressa*, on the other hand, this central spike is in a somewhat more posterior position along the length of the premaxillae. The spike process gives the ventral margin of the external naris a vague W-shape in anterior view.

Ventrally, the premaxilla form parts of the labial ridge (Fig. 5B). In FMNH UC 1501 and *Gopherus polyphemus*, the contact of the right and left premaxillae forms a sharp and ventrally protruding ridge in the skull midline, which is strongly outlined in former, rather weaker in the latter (Fig. 7B, C), and is entirely absent in *Manouria impressa* (Fig. 8B). *In Gopherus polyphemus*, the posterior end of the premaxillary part of the triturating surface also bears a transverse ridge that connects to the lingual ridge on the maxilla (Fig. 7B, C). This is absent in FMNH UC 1501 (Fig. 5B). At the posterior end of the premaxilla, the element forms the anterior margin of the foramen praepalatinum, which is posteriorly closed by the vomer in FMNH UC 1501 (Fig. 5B, E) and *Gopherus polyphemus* (Fig. 7B), but not in *Manouria impressa*, in which the foramen is completely ossified within the premaxilla (Fig. 8B).

### Maxilla

In FMNH UC 1501, both maxillae are preserved and nearly complete. The right element misses the tip of its posterior process, but this part is preserved in the left side (Fig. 5A–E). In UMMP 9318, the maxillae are also nearly complete, but more heavily fractured than in FMNH UC 1501 (Figs. 3C, D, 6A, B, D, E). The maxilla of *Stylemys nebrascensis* contacts the premaxilla anteriorly, the prefrontal dorsally and the jugal posteriorly. The medial margin is connected to the palatine, vomer, premaxilla and pterygoid (Figs. 5, 6). Such contacts can be similarly observed in both *Manouria impressa* and *Gopherus polyphemus*. However, in *Manouria impressa*, the maxilla is not in contact with the vomer (Fig. 8B).

The posterior process of the maxilla of *Stylemys nebrascensis* contributes to the ventral margin of the orbit, and extends posteriorly underneath the jugal to reach the anterior margin of the lower temporal emargination (Figs. 5C, 6D). The ascending process also contributes to the orbit, and anteriorly to the external naris. Posteromedially, the maxilla forms parts of the floor of the fossa orbitalis. Within this surface, there is a small foramen supramaxillare that extends internally into the canalis alveolaris superior. The canal opens anteriorly as the foramen alveolare superius, which is positioned in the medial maxillary surface near the anterior end of the nasal passage that leads into the nasal cavity. Ventrally, the maxilla forms the majority of the triturating surface.

The maxilla forms a deep and sharp-edged labial ridge in *Stylemys nebrascensis* (Figs. 5B, 6B). The triturating surface is transversely relatively broad, and in this regard more similar to the condition of *Gopherus polyphemus* than to the morphology of *Manouria impressa*. Again, as in *Gopherus polyphemus* (Fig. 7B, C), *Stylemys nebrascensis* has a prominent accessory ridge that parallels the course of the labial ridge, and which traverses nearly the entire maxillary triturating surface (Figs. 5B, 6B). The ridge levels off anteriorly, near the contact with the premaxilla. In *Gopherus polyphemus*, the accessory ridge instead reaches the premaxilla suture (Fig. 7B, C), whereas the accessory ridge is low and short in *Manouria impressa* (Fig. 8B). The lingual ridge of *Stylemys nebrascensis* is inconspicuous and low (Figs. 5B, 6B), which contrast with both the condition of *Gopherus polyphemus* (Fig. 7B, C), which has a ventrally deep lingual ridge, and the condition of *Manouria impressa*, which lacks a lingual ridge but is instead strongly ventrally bulged along the lingual margin of the triturating surface (Fig. 8B). The lingual ridge of *Stylemys nebrascensis* does not extend anteriorly to meet the interpremaxillary ridge (Fig. 5B), which is an important difference to all species of *Gopherus* (Fig. 7B, C). Considering that the lingual ridge of UMMP 9318 is low along its entire length, it seems likely that the specimen once had the same triturating morphology as FMNH UC 1501, which preserves the interpremaxillary ridge that is unfortunately not directly preserved in UMMP 9318.

### Vomer

The vomer is fully preserved in both specimens (Figs. 3B, 5B, 6B, C, E, F), but better so in FMNH UC 1501, as there is a large crack in the anterior vomer region of UMMP 9318. The vomer of *Stylemys nebrascensis* is a singular elongated and dorsally gently curved (Fig. 6C), median bone. The vomer is in contact with the premaxilla anteriorly, the maxilla anterolaterally, the prefrontal dorsally, the palatine laterally, the pterygoid posterolaterally, and the parabasisphenoid posteriorly.

The anterior part of the vomer is formed as an anteroventrally inclined columnar process, which forms the central bar between the internal nares (Figs. 5B, 6B). Anteriorly, at the contact with the premaxillae, the vomer of *Stylemys nebrascensis* forms the posterior margin of the foramina praepalatina (Fig. 5B). The vomer is not incorporated into the triturating surface. At the junction with the premaxillae and maxillae, the ventral surface of the vomer is smooth. This differs to all specimens of *Gopherus* spp. examined, in which there is a central depression, the anteromedial vomerine aperture of Crumly (1984). In *Gopherus polyphemus*, the cleft is developed as a narrow pit framed to either lateral side by sharp ridges (Fig. 7B, C). In *Gopherus agassizii* and *Gopherus flavomarginatus*, the cleft is deeper, penetrating the vomer completely to form a dorsoventral median opening just slightly posterior to the level of the foramina praepalatina (see Crumly, 1984: Fig. 3B).

The dorsolateral process of the vomer of *Stylemys nebrascensis* is in contact with the prefrontal (Fig. 6C), and the sulcus vomeri is formed on its dorsal surface. The vomer posteriorly becomes narrow and is situated between the palatines, where it forms a narrow dorsal trough posterior to the sulcus vomeri on the dorsal surface (Fig. 6F). The posterior tip of the vomer underlaps the elongated rostrum basisphenoidale of FMNH UC 1501, and contacts the pterygoid on the ventral skull surface (Fig. 5B). In UMMP 9318, the anterior elongation of the rostrum basisphenoidale beyond the level of the trabeculae is absent, resulting in a narrow absence of a vomer–parabasisphenoid contact (Fig. 6C). On the ventral surface of *Stylemys nebrascensis*, there is a prominent medial keel with a sharp margin, which extends along the entire ventral surface of the vomer, forming clearly separated nasal passages that are ventrally open (Figs. 5B, 6B). The keel of *Stylemys nebrascensis* is much more robust than in *Gopherus polyphemus* (Fig. 7B)*, Gopherus agassizii, or Gopherus flavomarginatus,* but still more similar to this taxon than to the low ridge seen in *Manouria impressa,* which fades along the posterior part of the ventral surface of the vomer (Fig. 8B).

### Palatine

The palatines are fully preserved in both specimens, but slightly fractured in UMMP 9318 (Fig. 6B), whereas the bones are pristine in FMNH UC 1501 (Fig. 5B, C, D). The palatine of *Stylemys nebrascensis* is a flat bone, forming the central parts of the palate. Its lateral process is in contact with the maxilla. Additionally, there is an anteromedial contact with the prefrontal, a posterodorsal contact with the parietal and a posterior contact with the pterygoid (Figs. 5B–D, 6B). Posterolaterally, at the level of the foramen palatinum posterius, there is a contact with the jugal, which can only be seen in dorsal view of the specimen. The palatine is not connected to its counterpart in the midline, as the vomer is positioned in the centre, completely separating them from one another (Figs. 5B, 6B). The palatine also does not contact the epipterygoid in *Stylemys nebrascensis* (Figs. 5D, 9).

The contacts of the palatine vary relatively strongly among *Stylemys nebrascensis*, *Gopherus polyphemus*, and *Manouria impressa*. For example, in contrast to *Stylemys nebrascensis*, the palatine does not contact the jugal at all in *Gopherus polyphemus or Manouria impressa*, as these taxa lack medial processes of the jugal*. Stylemys nebrascensis* and *Gopherus polyphemus* share the palatine–parietal contact, which is absent in *Manouria impressa*. *Stylemys nebrascensis* and *Manouria impressa*, on the other hand, share the absence of a palatine–epipterygoid contact, which is present in *Gopherus polyphemus* (Fig. 7D).

The palatine of *Stylemys nebrascensis* defines the parts of the floor of the fossa orbitalis, and the posterior margin of foramen orbito-nasale (Figs. 5C, D, 6B, D). The foramen platinum posterius is completely framed by the palatine, although the pterygoid extends into the margin of the foramen on the ventral skull surface (but not the dorsal one) (Figs. 5B, 6B). The transversely concave ventral surface of the palatine is contributing to the formation of the posterior margin of the internal naris. Posterodorsally, the palatine has an ascending process that takes part in the formation of the secondary lateral braincase wall (Figs. 5C, D, 6C, 9).

### Quadrate

The quadrate is only preserved on the left side of the FMNH UC 1501, where it is also damaged along the posterodorsal side (Fig. 5). UMMP 9318, however, preserves a basically complete, albeit slightly fractured right quadrate, whereas the bone is more severely damaged on the left side (Figs. 3, 6A, B, D, F). The quadrate of *Stylemys nebrascensis* contacts the prootic and the opisthotic medially, the quadratojugal anterolaterally, the squamosal posterolaterally, the pterygoid medially, and the epipterygoid anteriorly. The contact with the posterior tip of the epipterygoid in the lower temporal fossa is achieved via a short epipterygoid process (preserved only in FMNH UC 1501; Fig. 9A). The quadrate is not included in the trigeminal foramen margin, as the dorsal process of the pterygoid is blocking it (Fig. 9).

The quadrate of *Stylemys nebrascensis* forms the majority of the processus trochlearis oticum, which has a peculiar form in both specimens. The processus is developed as a near-circular peduncle with a concave anterodorsal surface (Figs. 5A, 6A, 9). We interpret this as a facet for an ossified os transiliens, a sesamoid ossification that occurs in the Oligocene extinct species *Oligopherus laticuneus* and extant gopher tortoises (Bramble, 1971, 1974; Hutchison, 1996; Ray, 1959). Bramble (1974) describes that the os transiliens is absent in *Stylemys nebrascensis*, but he also writes that “the presence of a distinct facet on the trochlear process of the quadrate seems to be a fairly reliable indicator of an osseous sesamoid in both fossil and Recent forms” (Bramble, 1974: p. 103).

The anterior surface of the quadrate of FMNH UC 1501 is similar to *Manouria impressa*, in that both species do not form a separate foramen from the canalis cavernosus (Figs. 6, 9), which likely is for the mandibular artery (e.g., Rollot et al., 2021a). Such a separate mandibular artery foramen is present, however, in all *Gopherus* species (Fig. 7D). Instead, *Manouria impressa* and *Stylemys nebrascensis* have posteroventrally elongated trigeminal foramina (see below), to which the quadrate does not contribute. This elongated morphology could reflect a coalescence between the trigeminal foramen and the mandibular artery foramen, or it could represent an early evolutionary stage in which these foramina have not yet separated as distinct openings.

The lateral surface of the quadrate of *Stylemys nebrascensis* is defined by a deep hollow cavity, the cavum tympani (Figs. 5C, 3C, D, 6D, 9). The cavum tympani of *Stylemys nebrascensis* is anteriorly fully formed by the quadrate, and the posterodorsal margin is also fully framed by the quadrate, as evident from the specimen UMMP 9318 (Figs. 6D, 9C). Thus, the quadrate fully circumscribes the antrum postoticum, forming a very large foramen posterodorsally into the antrum postoticum that can be best seen when the squamosal is digitally disarticulated. The same morphology is found in *Gopherus polyphemus* and *Manouria impressa*, although the squamosal reaches the margin of the cavum, overlying the posterodorsal quadrate in *Gopherus polyphemus* (Fig. 7D). The cavum tympani of *Stylemys nebrascensis* posterodorsally extends into a voluminous antrum postoticum (Fig. 9), again as in extant species of the *Gopherus*–*Manouria* group. The antrum postoticum and cavum tympani sensu stricto are roughly divided by a strong ridge in *Gopherus polyphemus* (Fig. 7C; and other *Gopherus* species), which extends from the incisura columella auris anterodorsally across the inner surface of the cavum tympani and which effectively creates a subcolumellar fossa in *Gopherus*. This ridge is absent in both *Stylemys nebrascensis* and *Manouria impressa*. The incisura columella auris is a tunnel-like opening for the stapes, and posteroventrally completely enclosed by the quadrate in *Stylemys nebrascensis, Manouria impressa,* and *Gopherus polyphemus.* Posterolaterally, the incisura is bordered by a strong ridge in *Stylemys nebrascensis,* which extends laterally to the posteroventral margin of the cavum tympani (Fig. 5C).

The mandibular condyle of *Stylemys nebrascensis* is a short, ventrally directed process that does not extend far ventrally beyond the level of the cavum tympani (Figs. 5C, 6C, 9). In contrast, the respective process on *Manouria impressa and Gopherus polyphemus* is more elongated, extends well beyond the ventral level of the cavum tympani, and is notably anteroventrally inclined (Figs. 8C, 7D). Another difference in the mandibular condyle of FMNH UC 1501 to turtles of the *Gopherus*–*Manouria* clade is that the articulation facet is medially expanded from the main body of the articular process, forming a small projecting lip (Fig. 5F). This lip effectively seems to displace the articulation surface for the mandible somewhat medially, which can be appreciated when the articulated lower jaw position is considered. The medial lip is not evident in UMMP 9318, and may thus be subject to ontogenetic variation, given the smaller size of UMMP 9318. In addition, the most lateral part of the ventral surface of the articular process in *Stylemys nebrascensis* seems to not be included in the jaw articulation. This lateral part instead forms a short, anteriorly projecting lamina, which is apparent as the expanded anteroventral margin of cavum tympani in lateral view (Figs. 5B). This lateral quadrate lamina is not present in *Gopherus polyphemus* or *Manouria impressa*, but we found the structure in many members of the Testudininae, in which the lamina is more strongly developed than in *Stylemys nebrascensis*, effectively forming an anterior sheet that conceals parts of the lower temporal fossa in lateral view (e.g., *Kinyxis erosa*: SMF 40166; *Testudo marginata*: FMNH 51672).

The quadrate of *Stylemys nebrascensis* also forms the lateral margin of the fenestra postotica, which leads into the cavum acustico-jugulare (Fig. 11A, B). Medially, within the bounds of the cavum acustico-jugulare, the quadrate forms the canalis cavernosus together with the prootic and pterygoid. Between the quadrate and the prootic, the canalis stapedio-temporalis connects the cavum acustico-jugulare with the adductor chamber. The foramen stapedio-temporale is the dorsal opening of the canalis stapedio-temporalis, formed between the quadrate and the prootic (Figs 3A, 5A, 6A). Below the ventral margin of the fenestra postotica, there is a highly unusual foramen and canal formed in the quadrate–pterygoid suture that can be confirmed in both specimens, although this region is much better preserved in FMNH UC 1501 (Figs. 5B, 6, 11A, B). The canal projects anterodorsally between both bones, and, for its most anterior part, is fully enclosed by the pterygoid. The canal has two dorsal openings. The first, more posterior one, is still situated in the quadrate and is confluent with the medial foramen of the incisura columella auris (Fig. 11B, D). The second, more anterior foramen already lies within the pterygoid, and enters the floor of the canalis cavernosus at the posterior end of the latter (Fig. 11D). We could not find a corresponding canal in any extant tortoise. The foramen and canal have a large diameter (Fig. 11B), so that we do not think that this canal could be for the chorda tympani ramus of the hyomandibular nerve, which may be expected to take such a course given it is a branch of the hyomandibular ramus of the facial nerve innervating the mandible. It seems possible that this is a canal for the lateral head vein itself, as this vessel is known to extend through the canalis cavernosus. This would imply that the lateral head vein of *Stylemys nebrascensis* does not enter the skull through the fenestra postotica as reported for other turtles. However, this is speculative. For the lack of a better name, we refer to the canal as the subcavernosal canal, and its posterior opening as the subcavernosal foramen and its dorsal pendants within the cranium as the dorsal subcavernosal foramina.

### Epipterygoid

The epipterygoid is fully preserved on both sides of FMNH UC 1501 (Figs. 5C, D, 9A). It is preserved but fractured in UMMP 9318 (Fig. 9C), so that the following morphological statements are entirely drawn from the FMNH specimen. The epipterygoid of *Stylemys nebrascensis* is a small, laminar ossification, surrounded largely by the pterygoid ventrolaterally and posterodorsally (Fig. 9A). The epipterygoid has only a small contact with the parietal anterodorsally, and only so on the right side (Fig. 5D), contrasting the broad contacts seen in *Gopherus polyphemus* and *Manouria impressa*. In addition, the epipterygoid of FMNH UC 1501 contacts the quadrate posteriorly, which is absent in *Gopherus polyphemus* and *Manouria impressa*. As in extant tortoises, the epipterygoid of FMNH UC 1501 does not contribute to the trigeminal foramen. Instead, it is positioned ventrally removed from its margin, and separated from the trigeminal foramen by a stout ridge formed by the pterygoid and parietal (Fig. 9). The epipterygoid is reduced in size in comparison to extant tortoises, but especially in comparison to *Gopherus polyphemus*: whereas the latter has a broad epipterygoid–palatine contact (Fig. 7D), the anterior extent of the epipterygoid in FMNH UC 1501 ends well before reaching the palatine (Fig. 9A). An epipterygoid–prootic contact, as present in *Manouria impressa*, is absent in FMNH UC 1501 (Fig. 9A).

**Fig. 10:**
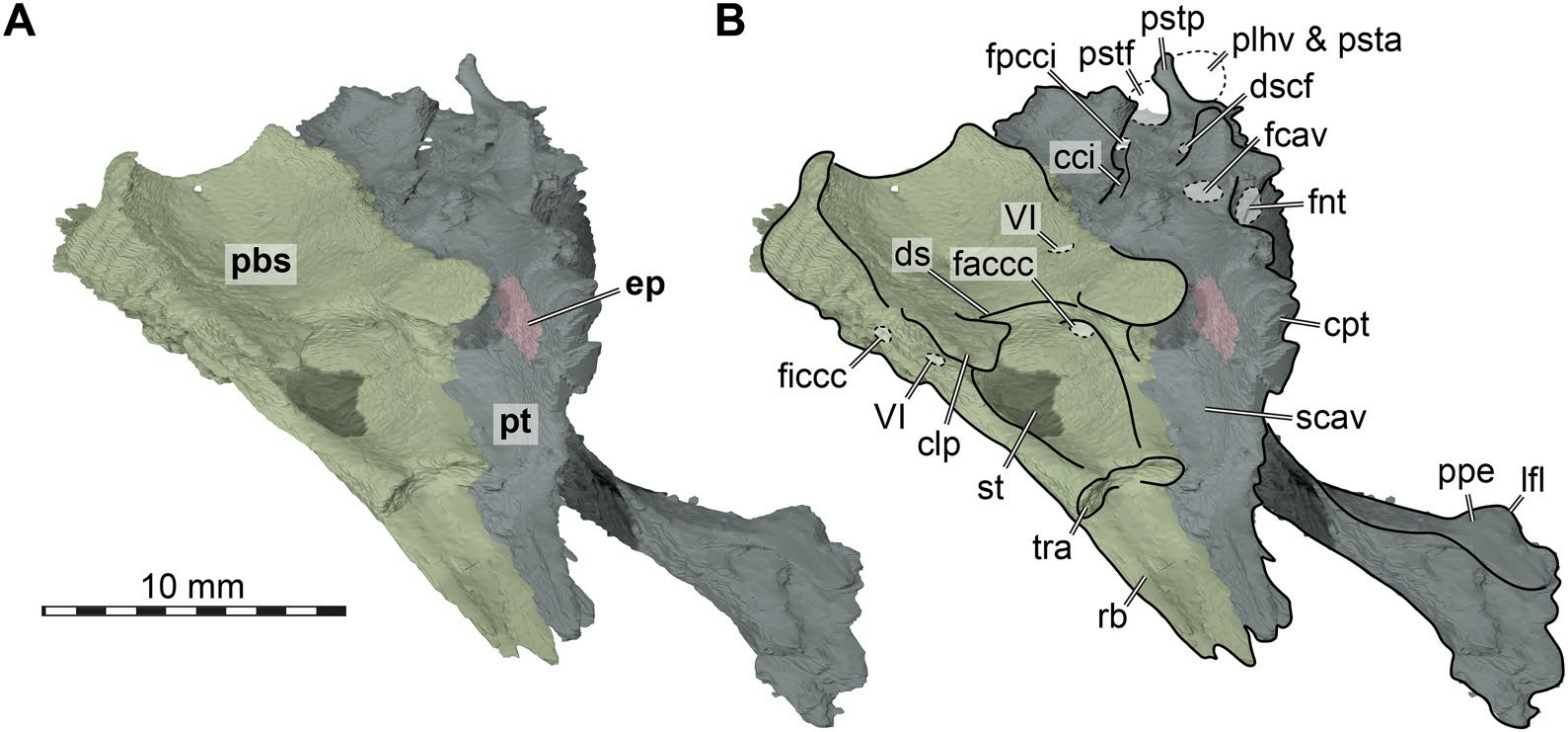
3D renderings of the parabasisphenoid, left pterygoid and left epipterygoid of FMNH UC 1501, *Stylemys nebrascensis*. **A** Left anterodorsolateral view. **B** Same as A, but with superimposed interpretative line drawing. Note that bones are labelled in bold font, and traits in regular font. Also note that many of the indicated foramina are only fully formed when the dorsally adjacent bones (prootic, opisthotic) are articulated. cci, canals carotici interni; clp, clinoid process; cpt, crista pterygoidei; ds, dorsum sellae; dscf, dorsal subcavernosal foramen; ep, epipterygoid; faccc, foramen anterius canalis carotici cerebralis; fcav, foramen cavernosum; ficcc, foramen internum canalis carotici cerebralis; fnt, foramen nervi trigemini; fpcci, foramen posterius canalis carotici interni; lfl, lateral flange; pbs, parabasisphenoid; plhv, passage for the lateral head vein; ppe, processus pterygoideus externus; psta, passage for the stapedial artery; pstf, poststapedial foramen; pstp, poststapedial process; pt, pterygoid; rb, rostrum basisphenoidale; scav, sulcus cavernosus; st, sella turcica; tra, trabecula; VI, abducens nerve foramen for the CN VI

Structurally, the epipterygoid of FMNH UC 1501 is minorly integrated into the secondary braincase wall: when the epipterygoid is digitally removed, there remains a small hole between the descending process of the parietal and the crista pterygoidei of the pterygoid, into which the epipterygoid fits (Fig. 10). As a result, the epipterygoid is expressed both in the medial and lateral surface of the secondary braincase wall, although the lateral surface is much larger than its medial exposure. The same is true for *Manouria impressa*, whereas the epipterygoid of *Gopherus polyphemus* is fully restricted to the lateral surface of the secondary braincase wall.

The posterior process of the epipterygoid in FMNH UC 1501 is posteriorly in contact with the epipterygoid process of the quadrate (Fig. 9A). This contact is prevented in *Gopherus polyphemus* and *Manouria impressa* by the fossa cartilaginis epipterygoidei.

### Pterygoid

The pterygoid of FMNH UC 1501 is fully preserved on the left side and incomplete on the right side, with the part of the posterior process that faces the quadrate being broken off (Fig. 5B). In UMMP 9318, both pterygoids are reasonably well preserved, but unfortunately, their dorsal surface is broken at the posterior end, as evidenced by the incomplete foramen posterius canalis carotici interni (Figs. 3B, 6B).

At the anterior end, the pterygoid of *Stylemys nebrascensis* is bifurcated into an anterolateral and an anteromedial process, which frame the posterior half of the palatine (Figs. 5B, 6B, 10). The anterolateral process extends anteriorly to contact the jugal and maxilla (Figs. 5B, 6B). G*opherus polyphemus* and *Manouria impressa* lack the pterygoid–jugal contact*,* due to the absence of medial process of the jugal (Figs. 8B, 7B). The lateral surface of the anterolateral process of *Stylemys nebrascensis* faces the subtemporal opening and is anteriorly expanded both laterally and dorsoventrally to form the transverse process (= processus pterygoideus externus) (Figs. 5B, 6B, 9). The dorsoventral expansion of this process forms a laminar flange, as in *Manouria impressa* (Fig. 8C) but in contrast to *Gopherus polyphemus*, in which the process lacks a lateral flange altogether. The medial side of the anterolateral process of the pterygoid of *Stylemys nebrascensis* barely frames the foramen palatinum posterius in ventral view (Figs. 5B, 6B), and not at all on the dorsal side. Instead, the foramen is largely bordered by the palatine and has a moderate size that is larger than a typical blood vessel opening. The anteromedial process of the pterygoid is shorter and less wide mediolaterally than the anterolateral process. It is inserted between the vomer and palatine, and tapers anteriorly (Figs. 5B, 6B, 10).

The pterygoid of *Stylemys nebrascensis* is mediolaterally constricted in its central part, posterior to the contact with the vomer. In this area, the pterygoid contacts its counterpart along the midline in ventral view (Figs. 5B, 6B). However, this contact is relatively short (shorter than the parabasisphenoid length). On the dorsal surface of FMNH UC 1501, the interpterygoid contact is completely absent (Fig. 10), as the parabasisphenoid separates both pterygoids in the midline. However, the contact is present in UMMP 9318 (Fig. 6C), due to the absence of a triangular anterior expansion of the rostrum basisphenoidale anterior to the level of the trabeculae (see parabasisphenoid). *Manouria impressa* and *Gopherus polyphemus* show both ventral and dorsal interpterygoid contacts.

**Fig. 11:**
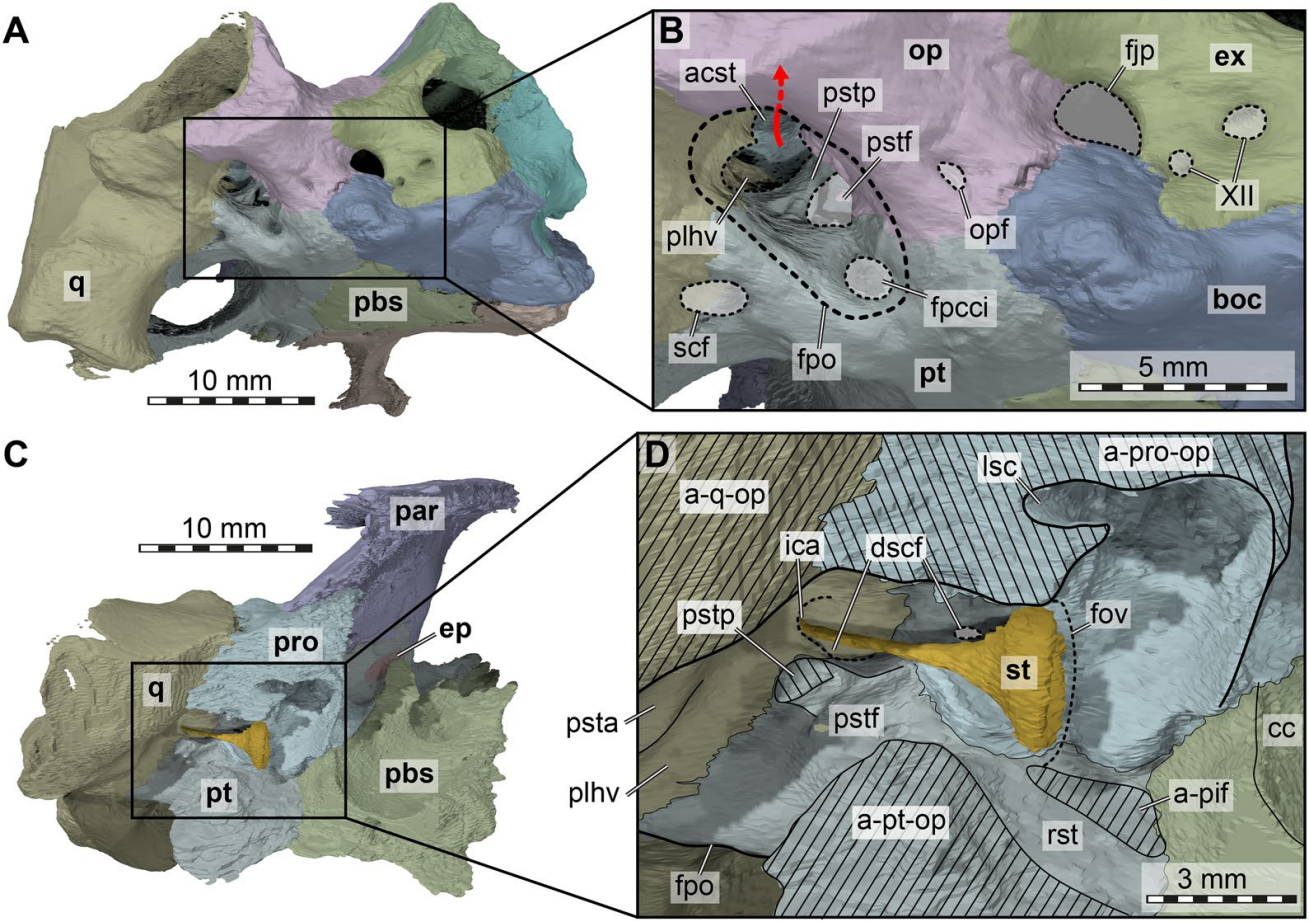
3D renderings of aspects of the occipital and cavum acustico-jugulare regions of FMNH UC 1501, *Stylemys nebrascensis*. **A** Posterior parts of the cranium in posterolateroventral view of the left side. **B** Close-up showing openings on the posterior skull surface with skull openings indicated as indicative line drawings. **C** Posterodorsomedial view onto partial posterior skull with the opisthotic, exoccipital and basioccipital removed. **D** Close-up of features of the cavum acustico-jugulare, inner ear, and recessus scalae tympani indicated as interpretative line drawings. Note that bones are labelled in bold font, and traits in regular font. Hatched bone surfaces indicate articulation surfaces of shown bones to those that were digitally removed to achieve the internal skull views. a-q-op, articulation surface of quadrate facing opisthotic; a-pif, articulation surface for the processus interfenestralis; a-pro-op, articulation surface of prootic facing opisthotic; a-pt-op, articulation surface of pterygoid facing opisthotic; acst, aditus canalis stapedio temporalis; boc, basioccipital; cc, cavum cranii; dscf, dorsal subcavernosal canal foramina; ep, epipterygoid; ex, exoccipital; fov, fenestra ovalis; fpcci, foramen posterius canalis carotici interni; fpo, fenestra postotica; fjp, foramen jugulare posterius; ica, incisura columellae auris; op, opisthotic; opf, oropharyngeal foramen; par, parietal; pbs, parabasisphenoid; plhv, path of lateral head vein; pro, prootic; psta, path for the stapedial artery; pstf, poststapedial foramen; pstp, poststapedial process; pt, pterygoid; q, quadrate; rst, recessus scalae tympani; scf, subcavernosal foramen; st, stapes; XII, foramina for the hypoglossal nerve (CN XII)

The pterygoid of *Stylemys nebrascensis* diverges laterally from the midline at about mid-length and frames the parabasisphenoid medially along its full length with its posterior process, which extends to the back of the skull where it forms an extensive contact with the basioccipital (Figs. 5B, 6B). However, a contact with the exoccipital is absent (Figs. 5F, 6F, 11A). The posterior process of the pterygoid is laterally expanded to form a process that abuts the medial side of the quadrate. The pterygoid fossa that is generally located on the ventral surface of this area is extremely shallow (Figs. 5B, 6B), yet deeper than in extant turtles of the *Manouria*–*Gopherus* clade, in which this surface is flat (Figs. 8B, 7B). *Stylemys nebrascensis* has a week ridge on the ventral pterygoid surface that likely indicates the course of muscle attachment. The ridge extends nearly along the entire ventral surface of the pterygoid, and becomes stronger and more robust in the constricted mid-part of the pterygoid. More anteriorly, it extends along the pterygoid–palatine contact (Figs. 5B, 6B). These ridges are more strongly developed in the larger specimen, FMNH UC 1501.

Within the suture to the quadrate and close to the margin of the fenestra postotica, *Stylemys nebrascensis* bears the subcavernosal foramen that opens into the subcavernosal canal (Figs. 5B, 11A, B). As described above (see quadrate), this canal extends anterodorsally and opens in two places, the more anterior one of which is formed by the pterygoid at the posterior end of the floor of the canalis cavernosus (Fig. 11B, D). This anterodorsal opening of the canal lies entirely within the pterygoid.

The posterior end of the pterygoid forms a posteriorly concave margin to the fenestra postotica of *Stylemys nebrascensis*. Just dorsally above this margin, the pterygoid of FMNH 1501 encloses the foramen posterius canalis carotici interni (fpcci) for the entry of the interna carotid artery (Fig. 11A, B). This will have been the same in UMMP 9318, in which this area is broken but in which the ventral outlines of the foramina are still present. The complete pterygoid encasing of the fpcci differs from the generalized testudinoid condition that is also seen in *Gopherus polyphemus* and *Manouria impressa*, in which the entry for this blood vessel starts as a dorsally open groove in floor of the cavum acustico-jugulare and becomes only covered intracranially by the prootic (Rollot et al., 2021a). However, more anterior parts of the internal carotid canal are also dorsally roofed by the prootic in FMNH 1501. Anteromedially, the internal carotid canal exits the pterygoid and enters the parabasisphenoid as the canal for the cerebral artery (Rollot et al., 2021a). A lateral branch or canal for the palatine artery is not expected in testudinids (Rollot et al., 2021a), and seems to be generally absent in *Stylemys nebrascensis* based on UMMP 9318 and the left side of FMNH UC 1501. The right side, however, does show a canal that branches off the internal carotid canal, and which opens in the floor of the sulcus cavernosus, thus fulfilling the topological expectations for a palatine artery canal. Interestingly, our specimen of *Manouria impressa* shows the same right–left asymmetry (see Rollot et al., 2021a: Fig. 17), with a potential palatine artery canal formed on the right side, but not on the left.

The fenestra postotica as the opening into the cavum acustico-jugulare is relatively small and far lateralized in *Stylemys nebrascensis*, which is due to the robust ventral process of the opisthotic, which separates the opening from the foramen jugulare posterius (Fig. 11A, B). Within the cavum acustico-jugulare, the dorsal surface of the pterygoid of FMNH UC 1501 (broken in UMMP 9318) bears a columnar process that contacts the opisthotic (Figs. 11B, D, 10). This process is highly unusual for turtles, and we herein refer to it as the ‘poststapedial process’, in reference to its topological position immediate posterior to the trajectory of the stapes, which traverses the cavum acustico-jugulare mediolaterally from the incisura columellae auris laterally to the fenestra ovalis medially. The poststapedial process of the pterygoid effectively partitions the cavum acustico-jugulare: The space lateral to the poststapedial process connects the fenestra postotica anteroposteriorly with the canalis cavernosus and the canalis stapedio temporalis (Fig. 11B). Medially, the poststapedial process and the opisthotic form a foramen within the cavum acustico-jugulare that connects the fenestra postotica region with the space just lateral to the fenestra ovalis as well as the lateral opening into the recessus scalae tympani (Fig. 11B, D). We use the term ‘poststapedial foramen’ herein for this opening. The poststapedial foramen is certainly unusual for turtles and, to our knowledge, has also not been described for testudinids so far. Nevertheless, we found poststapedial foramina (and processes) it in *Chelonoidis* sp. (SMF 67582), *Indotestudo elongata* (SMF 1585), *Indotestudo forstenii* (SMF 73257), *Homopus areolatus* (OUMNH 9403), *Testudo horsfieldii* (OUMNH 10344, PCHP 2929), and *Testudo marginata* (FMNH 51672) among our comparative sample of extant testudinids. It is absent, however, in many other testudinids, including species of the *Gopherus*–*Manouria* clade. Thus, this morphology seems to be widespread but not ubiquitous among testudinids. Importantly, the presence of this foramen in extant representatives of the group will allow testing which types of structures pass through the foramen. Due to its topological position, it seems likely that it would house structures that enter the recessus scalae tympani that do not already pass through the foramen jugulare posterius, and contrast-enhanced soft-tissue scans will be able to test this in future work.

The dorsal surface of the pterygoid of *Stylemys nebrascensis* forms a small part of the ventral floor of the endosseous labyrinth, as the opisthotic and prootic do not fully overlay the pterygoid in this area (Fig. 11D). Within this floor of the labyrinth cavity, the pterygoid has a small surface that articulated with the processus interfenestralis of the opisthotic (Fig. 11D). At the anterior end of the cavum tympani, the pterygoid of *Stylemys nebrascensis* forms the floor of the canalis cavernosus. At the anterior end of the canalis cavernous, the pterygoid forms the vertically laminar crista pterygoidei, which generally increases in height anteriorly (Fig. 10). The posterior part of the dorsal margin of the crista pterygoidei forms the ventral margin of the trigeminal foramen, and the pterygoid forms a short dorsal ramus at the posterior end of the trigeminal foramen (Fig. 9). This foramen is otherwise bound by the prootic and parietal (Fig. 9). The ventral margin of the trigeminal foramen as formed by the pterygoid and parietal protrudes slightly laterally to form a subtrigeminal ridge, which borders an overhanging lateral surface of the pterygoid onto which the small epipterygoid articulates (Fig. 9). When the epipterygoid is prepared away digitally, the lateral surface of the pterygoid is deeply recessed and bears an opening through which the epipterygoid inserts to become visible on the medial surface of the secondary braincase wall (Fig. 10). The epipterygoid is thus entirely circumscribed by the pterygoid bone in FMNH 1501. This differs to the more extensive contacts the epipterygoid shows in *Gopherus polyphemus* or *Manouria impressa* (see epipterygoid). Ventrally to the epipterygoid position, the lateral surface of the pterygoid of *Stylemys nebrascensis* bears a small foramen for the exiting vidian nerve canal (Fig. 9). This differs from *Gopherus polyphemus* and *Manouria impressa*, in which the vidian canal extends through the pterygoid and into the palatine, from which it exits in a comparatively further anterior position between the anterior end of the secondary lateral braincase wall and the foramen palatinum posterius (see also Rollot et al., 2021a). The crista pterygoidei of *Stylemys nebrascensis* is dorsally overlain by the parietal, and anteriorly bordered by the ascending process of the palatine (Fig. 9).

The pterygoid of *Stylemys nebrascensis* forms the floor of the sulcus cavernosus, which is the anterior extension of the canalis cavernosus and which is bordered by the raised surfaces of the parabasisphenoid medially and the crista pterygoidei laterally (Fig. 10). FMNH 1501 shows an interesting cranial asymmetry in this region, which was also observed and described for a specimen of *Manouria impressa* by Rollot et al., (2021a: Fig. 17): the right pterygoid of FMNH 1501 has a foramen in the floor of the canalis cavernosus that opens into the internal carotid system. Its position and size are consistent with an identification for the palatine artery (i.e. foramen anterius canalis carotici palatinum of Rabi et al., 2013; or foramen anterius canalis carotici lateralis of Rollot et al., 2021a), although testudinids generally lack the presence of the palatine artery (Rollot et al., 2021a). It is interesting that two turtles of the same clade show the same right–left asymmetry, although the significance of this is currently unclear. Another interesting feature of the pterygoid of FMNH 1501 in the sulcus cavernosus region is that it forms the lateral margin of the anterior abducens nerve foramina (Fig. 10). This differs from UMMP 9318, *Manouria impressa* and *Gopherus polyphemus*, in which the abducens foramina are fully formed within the parabasisphenoid. A contribution of the pterygoid to this foramen is generally rare in turtles, but has recently been observed for a range of paracryptodires (Evers et al., 2020: *Pleurosternon bullockii*; Evers et al., 2021: *Arundelemys dardeni*; Rollot et al., 2021b: *Uluops uluops*).

### Supraoccipital

The supraoccipital is incompletely preserved in both specimens of *Stylemys nebrascensis*. Whereas the central part of the supraoccipital is preserved in FMNH UC 1501, the supraoccipital crest is incompletely preserved and the posterior tip in the fossil is composed of sediment, which may have been modelled together with plaster or which may be a pseudomorphic infill of the original crest (Figs. 4, 5A, F). In UMMP 9318, a short part of the supraoccipital crest is preserved, but the majority of the process is also broken off (Fig. 6A, C, F). The supraoccipital of *Stylemys nebrascensis* contributes to the formation of the dorsal roof of the cavum cranii and foramen magnum. It contacts the prootic and the opisthotic ventrolaterally and the exoccipital posteroventrally. Anterodorsally, the supraoccipital is overlaid by the parietal.

As in *Manouria impressa* and *Gopherus polyphemus*, the overlapping posterior process of the parietals extend posteriorly far back, up to the level of the foramen magnum, so that the supraoccipital of *Stylemys nebrascensis* in not expressed in the skull roof surfaces (Figs. 5A, 6A).

Within the otic capsule of *Stylemys nebrascensis*, the supraoccipital is forming a triple junction with the prootic anteriorly and the opisthotic posteriorly (Figs. 5A, 6A). Internally, the supraoccipital contributes to forming the inner ear cavity. The inner ear cavity is open toward the cavum cranii via a large hiatus acusticus (Fig. 6C), as in most turtles. The supraoccipital hereby forms the dorsal margin of the hiatus acusticus. In this margin is a large, concave, semicircular notch within the medioventral wall of the cavum labyrinthicum of FMNH UC 1501, which is not evident in UMMP 9318. Although this notch could include the foramen aquaducti vestibuli, the latter is usually an extremely small foramen (e.g., Gaffney, 1972). Moreover, our segmented specimen of *Gopherus polyphemus* clearly shows a small foramen aquaducti vestibuli as well as the semicircular notch. The notch, however, is absent in *Manouria impressa*.

### Exoccipital

The exoccipital is fully preserved on the left side, but only partially on the right side in FMNH UC 1501 (Fig. 5F), whereas it is complete on both side of UMMP 9318 (Figs. 3F, 6C, F). The exoccipital forms the lateral margin of the foramen magnum. Its anterolateral surface contacts the opisthotic, and there is a dorsomedial contact with the supraoccipital (Figs. 5F, 6C, F).

The ventral part of the exoccipital forms an anteroposteriorly and mediolaterally expanded foot-like process that is contacting the basioccipital (Fig. 6C). The anterior part of the process is dorsally recurved with its anterior tip below the foramen jugulare anterius, contacting the opisthotic in FMNH UC 1501, whereas the ventral part of the opisthotic is not completely enough preserved in UMMP 9318 to verify this contact. The posterior end of the exoccipital footplate forms contributes to the formation of the condylus occipitalis, which is formed in roughly equal parts by both exoccipitals and the basioccipital (Figs. 5F, 6F).

The right and left exoccipital have a midline contact in the basioccipital condyle that extends anteriorly to the level of the foramen magnum. Along the dorsal ramus that frames the top part of the foramen magnum, the exoccipital bears a relatively robust neurapophyseal ridge (Figs. 5F, 6F). The occipital surface of the exoccipital, at the base of the ventral process is pierced by two canals for hypoglossal nerve (CN XII) (Fig. 11A, B). The exoccipital forms the medial margin of the foramen jugulare posterius, which is distinct from the fenestra postotica. A contact with the pterygoid is absent (Fig. 11A, B).

### Basioccipital

The basioccipital is fully preserved in FMNH UC 1501 and UMMP 9318 (Figs. 5B, F, 6B, C, F). It is located at the posterior end of the skull midline, and is mediolaterally broader than it is anteroposteriorly long. The basioccipital contributes to the floor of the cavum cranii, the occipital condyle, the margin of the foramen jugulare posterius, and forms the tuberculae basioccipitalis. It contacts the parabasisphenoid anteriorly, the pterygoid anterolaterally, the exoccipital dorsally, and the opisthotic laterally.

The lateral ends of the basioccipital of *Stylemys nebrascensis* project somewhat posteriorly, forming the tuberculae basioccipitalis (Figs. 5B, 6B). The tuberculae are more pronounced in FMNH UC 1501 than in UMMP 9318. The ventral surface between both tuberculae is gentry recessed and smooth. The posterior margin of the basioccipital narrows posteromedially to form a short, necked posterior process that forms the base of the occipital condyle (Figs. 5B, 6B). The posterior margin has a small posterior projection halfway between the main basioccipital tubercle and the occipital condyle in FMNH UC 1501, whereas there is simply a ridge between both structures in UMMP 9318. Above this posterior margin, the basioccipital of both specimens contributes to the occipital surface below the level of the foramen jugulare posterius, which is framed by the basioccipital, exoccipital, and opisthotic (Figs. 5F, 6F, 11B).

The dorsal part of the basioccipital is wedged between the exoccipitals and parabasisphenoid and only a small, triangular surface of the basioccipital contributes to the floor of the cavum cranii. This is similar to gopher tortoises and *Manouria impressa*. *Stylemys nebrascensis* bears a low and inconspicuous ridge, the crista dorsalis basioccipitalis. The ridge is restricted to a short anteromedian portion of the basioccipital, and is not anteriorly accompanied by a basis tuberculi basalis. Posterior to its contact with the processus interfenestralis of the opisthotic, the basioccipital forms the majority of the floor of the recessus scalae tympani. This surface is medially strongly recessed below the level of the foramen jugulare anterius, so that the basioccipital bears two ventrally deeply penetrating lateral fossae in dorsal view. These pockets are also present in *Manouria impressa* and *Gopherus polyphemus*.

### Prootic

The prootic is fully preserved on the left side and only partially on the right side in FMNH UC 1501 (Figs. 5A, D, 9A, 11D). The prootics are preserved on both sides of UMMP 9318, but they are partially fractured (Figs. 6A, C, 9C). The prootic contacts the quadrate laterally, the opisthotic posteriorly, the supraoccipital posteromedially, the descending process of the parietal anterodorsally, and the parabasisphenoid ventromedially. The prootic forms the anteromedial part of the otic capsule, the dorsal and medial margin of the canalis cavernosus, the dorsal margin of the foramen cavernosus and the foramen nervi trigemini, the medial margin of the foramen stapedio-temporale and the medial half of the processus trochlearis oticum.

The prootic of *Stylemys nebrascensis* has a reduced exposure within the floor of the adductor chamber (Figs. 5A, 6A) compared to gopher tortoises (Fig. 7A). This is in part caused by an anteroventral expansion of the parietal, which overlaps parts of the prootic within the floor of the adductor fossa. The reduced exposure is, however, also apparent in *Manouria impressa* (Fig. 8A). The dorsal surface of the prootic of *Stylemys nebrascensis* bears the foramen stapedio-temporalis, which is large and leads ventrally into the cavum acustico-jugulare (Figs.  3A, 5A, 6A). At the anterior end of the otic process, the prootic contributes only minorly to the processus trochlearis oticum, which projects anteriorly and is very well-developed (Figs. 5A, 6A, 9B, D). The resulting anteroventrally exposed surface of the prootic contributes to the trigeminal foramen, which is posteriorly elongated to include a confluent ‘foramen’ for the mandibular artery (Fig. 9; e.g., Gaffney, 1979; Evers & Benson, 2019; Rollot et al., 2021a). This is as in *Manouria impressa* but unlike the condition of gopher tortoises, in which all species for which we have digital data (Fig. 7D; i.e. *Gopherus polyphemus*, *Gopherus agassizii*, *Gopherus flavomarginatus*) have a less posteriorly elongated trigeminal foramen, which is separated from a separate mandibular artery foramen.

The prootic of *Stylemys nebrascensis* composes a foot-like process that is medioventrally contacting the parabasisphenoid and laterally the pterygoid (Fig. 11D). This process is posteriorly well-developed, and forms parts of the floor of the cavum labyrinthicum. The prootic also makes up the anterior half the hiatus acusticus and the semicircular canal system, and the anterior margin of the fenestra ovalis (Figs. 5D, 6C, 11D). The fenestra ovalis is not fully circumscribed by the prootic and opisthotic, but remains ventrally ‘open’ toward the braincase floor formed by the pterygoid. The posteriorly facing surface of the prootic that is within the cavum acustico-jugulare and immediately lateral to the fenestra ovalis shows a prootic recess as described for some other turtles (e.g., Evers & Benson, 2019), although this is less deeply or spaciously developed as in some other turtles (e.g., Evers & Joyce, 2020). The lateral semicircular canal of the labyrinth is apparent as a groove within the prootic (Fig. 11D), but only the portion in the opisthotic is medially fully ossified to a canal. The fossa acustico-facialis is embedded into the medial surface of the ventral process of the prootic (Fig. 6C). From here, three small foramina for the acoustic nerve can be observed in both specimens. Additionally, the facial nerve canal extends through the ventral process towards the canalis cavernosus. In the floor of the canalis cavernous, situated in the suture of the prootic with the pterygoid, there is a small, medioventrally directed canalis pro ramo nervi vidiani which carries the vidian nerve into the internal carotid canal, and which is only visible in the better preserved FMNH UC 1501. The geniculate ganglion is inferred to be positioned within the canalis cavernosus.

### Opisthotic

The opisthotic of FMNH UC 1501 is near complete on the left side, with the lateral end of the paroccipital process being broken off (Fig. 5A, F). On the right side, the lateral part of the opisthotic is broken off, revealing a clear view onto the processus interfenestralis and recessus scalae tympani (Fig. 5D), which are hard to observe in complete specimens. In UMMP 9318, the left prootic is laterally broken, but the right one is reasonably complete, with damage only near the fenestra postotica region (Figs. 3F, 6A, C, F). The opisthotic of *Stylemys nebrascensis* contacts the prootic anteriorly, the supraoccipital mediolaterally, the pterygoid ventrally, the quadrate laterally, the basioccipital posteroventrally, and the exoccipital posteromedially. A contact with the squamosal is narrowly absent in UMMP 9318 (Fig. 6F), and this cannot be accessed in FMNH UC 1501 in which both opisthotics are laterally not complete enough to examined their extent. The opisthotic of *Stylemys nebrascensis* forms the posterior part of the inner ear cavity, the processus interfenestralis, contributes to the fenestra ovalis, the fenestra perilymphatica, the foramen jugulare anterius and posterius, the fenestra postotica, the oropharyngeal foramen, and the recessus scalae tympani.

The dorsal surface of the opisthotic is broadly exposed within the floor of the upper temporal fossa, between the exoccipital, supraoccipital, prootic and quadrate (Figs. 5A, 6A). There is a low but distinct ridge that trends from the anteromedial end of the surface posterolaterally in FMNH UC 1501 (Fig. 5F). Together with a roughly parallel ridge on the exoccipital, this ridge of the opisthotic defines a shallow fossa between both bones, a feature that is neither developed in *Gopherus polyphemus*, nor *Manouria impressa*. In UMMP 9318, both ridges and the fossa are less clearly developed, but also present (Fig. 6F). The paroccipital process, only fully preserved in UMMP 9318, is broad and laterally short. It is a few millimetres shy of contacting the squamosal (Fig. 6F), whereas this contact is relatively broadly present in *Manouria impressa* and *Gopherus polyphemus* (Figs. 8D, 7E).

The paroccipital process of *Stylemys nebrascensis* is ventrally buttressed by a massive ventromedially directed process of the opisthotic that contacts the pterygoid and basioccipital (Figs. 5F, 6F, 11B). This ventral opisthotic process separates the foramen jugulare posterius medially from the more laterally positioned fenestra postotica (Fig. 11B). Centrally between both openings, the ventral process of the opisthotic has an unusual foramen (Fig. 11B). The foramen is small as is typical for smaller nerve foramina, and leads into a canal that opens within the recessus scalae tympani. We found similar foramina in all examined gopher tortoises (i.e. *Gopherus polyphemus*, *Gopherus agassizii*, *Gopherus flavomarginatus*) as well as *Manouria impressa*, although the foramina in these turtles are sometimes much wider and sometimes incompletely separated from the foramen jugulare posterius (e.g., Fig. 8D). Gaffney (1979: Fig. 98) labelled a respective foramen in *Gopherus polyphemus* with a question mark, indicating that the identity of this unusual foramen was unclear. We also found the foramen in *Kinyxis erosa* (SMF 40166), *Chelonoidis* sp. (SMF 67582), *Indotestudo elongata* (SMF 1585), *Indotestudo forstenii* (SMF 73257), *Homopus areolatus* (OUMNH 9403), *Psammobates tentorius* (IW 1149), *Testudo horsfieldii* (OUMNH 10344, PCHP 2929), *Testudo marginata* (FMNH 51672), but not in *Aldabrachelys gigantea* (NHMUK 77.11.12.2) or *Malacochersus tornieri* (SMF 58702). The foramen can be roughly aligned with the medial, internal, and external glossopharyngeal foramina (described below) of FMNH UC 1501 and UMMP 9318, and thus we think that the unusual canal is for rami of the glossopharyngeal nerve. Shiino (1912) describes how the glossopharyngeal nerve swells to form the ganglion petrosum upon exiting the external glossopharyngeal foramen on the lateral surface of the processus interfenestralis. Two main posterior rami emerge from there, the ramus pharyngeus and the ramus lingualis (Shiino, 1912). These would usually exit the recessus scalae tympani through the fenestra postotica, but it seems that *Stylemys nebrascensis* and closely related tortoises have specific foramina for these nerves. As both nerves innervate parts of the mouth region (Shiino, 1912), the tongue, and the tongue bones, we call the opening “oropharyngeal foramen” herein.

The opisthotic of *Stylemys nebrascensis* forms the dorsal border of the fenestra postotica (Fig. 11B), whereas the floor is formed by the pterygoid and the quadrate contributes to the lateral margin. Shortly within the opening and thus already within the cavum acustico-jugulare, there is an unusual dorsal process of the pterygoid (see ‘poststapedial process’ in the pterygoid description) that makes contact dorsally with the opisthotic (Fig. 11B). By this bony connection, the cavum acustico-jugulare becomes effectively partitioned. The space lateral to the poststapedial process leads toward the canalis cavernosus and the canalis stapedio-temporalis. Medially, the poststapedial process and the opisthotic form an internal foramen that leads toward the space just before the fenestra ovalis, and which we term ‘poststapedial foramen’ herein (Fig. 11B, D). Whereas we could not find this foramen in most extant comparative material (including gopher tortoises and *Manouria impressa*), we did find it in *Chelonoidis* sp. (SMF 67582), *Indotestudo elongata* (SMF 1585), *Indotestudo forstenii* (SMF 73257), *Homopus areolatus* (OUMNH 9403), *Testudo horsfieldii* (OUMNH 10344, PCHP 2929), and *Testudo marginata* (FMNH 51672).

The processus interfenestralis of *Stylemys nebrascensis* is a delicate process that diverges ventrally from the opisthotic, enclosing the cavum labyrinthicum posteriorly and forming the anterior wall of the recessus scalae tympani (Figs. 5D, 6C). Its ventral margin is somewhat expanded and has a contact with the dorsal surface of the pterygoid (Fig. 11D). This is similar to the condition in *Manouria impressa*, but unlike the condition of gopher tortoises, is which the ventral surface of the processus interfenestralis is not covered by the pterygoid, thus exposing parts of the cavum acustico-jugulare (Fig. 7B). As in the extant relatives, FMNH UC 1501 has a contact with the exoccipital along the ventromedial end of the processus interfenestralis, thereby fully enclosing the foramen jugulare anterius. The ventral contact of the processus interfenestralis with the pterygoid, and the contact with the exoccipital in the margin of the foramen jugulare anterius are not seen in UMMP 9318, as the specimen overall seems less ossified than FMNH UC 1501. A contact of the opisthotic with the basioccipital is not seen in either of our specimens of *Stylemys nebrascensis*, contrary to the condition in *Manouria impressa,* but mirroring the condition in *Gopherus polyphemus*. Anterior to the foramen jugulare anterius, the medial margin of the processus interfenestralis of FMNH UC 1501 bears an incomplete medial glossopharyngeal foramen (sensu Gaffney, 1972, 1979), which is again not ossified in UMMP 9318. The glossopharyngeal nerve enters the cavum labyrinthicum through this foramen in FMNH UC 1501. The opisthotic is then pierced by a short canal, starting medially from within the cavum labyrinthicum and existing the processus interfenestralis at its base into the recessus scalae tympani. This external glossopharyngeal foramen can be seen in both specimens. Upon exiting this foramen, the nerve would from its ganglion (Shiino, 1912). The processus interfenestralis of *Stylemys nebrascensis* is also anteroposteriorly pierced by a larger opening, the fenestra perilymphatica. This opening between the cavum labyrinthicum and recessus scalae tympani is otherwise ventrally bound by the basioccipital, and medially by the exoccipital. Along its lateral margin, the processus interfenestralis frames the fenestra ovalis posteriorly but does not extend further to contact the prootic, leaving the fenestra ovalis fully open ventrally (Figs. 5D, 6C).

### Parabasisphenoid

The parabasisphenoid is completely preserved in both specimens examined for *Stylemys nebrascensis* (Figs. 5B, 6B, C, 10). It contacts the pterygoid, the basioccipital, the prootic, and also has an extensive contact with the vomer (see below). A contact with the exoccipital is absent, but just by a few millimetres in the more mature specimen FMNH UC 1501, whereas a contact with the opisthotic is definitely absent.

The ventral surface of the parabasisphenoid forms an anteriorly tapering triangle that is wedged between the pterygoids, which anteriorly separate the element from the vomer (Figs. 5B, 6B). The dorsal exposure of the parabasisphenoid is much larger, at least in FMNH UC 1501. In this specimen, the rostrum basisphenoidale has two parts (Fig. 10). The posterior part of the parabasisphenoid is dorsally excavated by the fossa for the pituitary, and laterally framed by the trabeculae. Whilst this is the same in both specimens, FMNH UC 1501 has a second part of the rostrum, which extends anteriorly beyond the level of the trabeculae as a tapering, triangular, flat sheet (Fig. 10). This sheet is not exposed ventrally, and rather overlapped by the ventromedial contact of the pterygoid with its counterpart. The anterior part of the rostrum basisphenoidale of FMNH UC 1501 contacts the vomer within the cranial cavity. This anterior process is entirely absent in UMMP 9318 (Fig. 6C). Instead, this specimen has the morphology that can also be observed in *Manouria impressa* and *Gopherus polyphemus*, in which no dorsal contact between the parabasisphenoid and vomer are observed. In *Manouria impressa*, this is caused by the effective absence of a rostrum basisphenoidale beyond the anterior end of the trabeculae, as in UMMP 9318. In *Gopherus polyphemus*, a short rostrum does extend beyond the anterior end of the trabeculae, but it is much shorter than in FMNH UC 1501*.*

In the dorsal view of all three testudinid species, the trabeculae form the medial margin to the sulcus cavernosus and frame the sella turcica, which is located on the dorsal surface of the rostrum basisphenoidale as a deep depression for the pituitary within the posterior end of the process (Fig. 10). The foramina anterior canalis carotici basisphenoidalis are located in the posterolateral aspects of the sella turcica and are extremely widely spaced in *Stylemys nebrascensis* (Fig. 10), whereas they are closer to one another in *Manouria impressa* and *Gopherus polyphemus*. The dorsum sellae of *Stylemys nebrascensis* is a low, transverse, step-like ridge between the clinoid processes. As a consequence of the low morphology of the dorsum sellae, the posterodorsal surface of the parabasisphenoid that holds the hindbrain slopes vertically into the sella turcica (Figs. 6C, 10). The degree of this slope is similar in *Manouria impressa*, but even lesser in *Gopherus polyphemus*, which has a particularly low dorsum sellae. The foramen anterius nervi abducens is positioned at the base of the clinoid process in *Stylemys nebrascensis* (Fig. 10). The foramen is formed in the pterygoid–parabasisphenoid sutures in FMNH UC 1501, whereas it is contained fully within the parabasisphenoid in UMMP 9318, *Manouria impressa* and *Gopherus polyphemus*. The latter has clearly developed retractor bulbi pits, as the base of the clinoid process is supported by a vertical lamina of bone, which projects anteriorly along the trabecula. *Stylemys nebrascensis* and *Manouria impressa* lack this lamina as well as clearly defined retractor bulbi pits. The clinoid processes of all three testudinids have very broad bases unlike those seen in most other non-testudinid turtles. In *Stylemys nebrascensis* and *Gopherus polyphemus*, the tips of the processes become even broader, forming an anteriorly projecting flat surface that is similarly shaped to prezygapophyses of vertebrae (Fig. 10).

The posterior part of the parabasisphenoid of *Stylemys nebrascensis* forms the typical, cup-shaped depression for the hindbrain. Along the lateral margin of the parabasisphenoid cup, the bone contacts the prootic anteriorly. Posterior to this contact, the parabasisphenoid contributes to the formation of the ventral margin of the hiatus acusticus, but a contact with the opisthotic posterior to this is absent. The posterior contact with the basioccipital is straight and flat for most of the contact (Fig. 6C), and a small gap between both bones remains. However, ventrally, the parabasisphenoid forms a thin sheeted layer of bone that slightly underlaps the basioccipital.

### Stapes

**Fig. 12:**
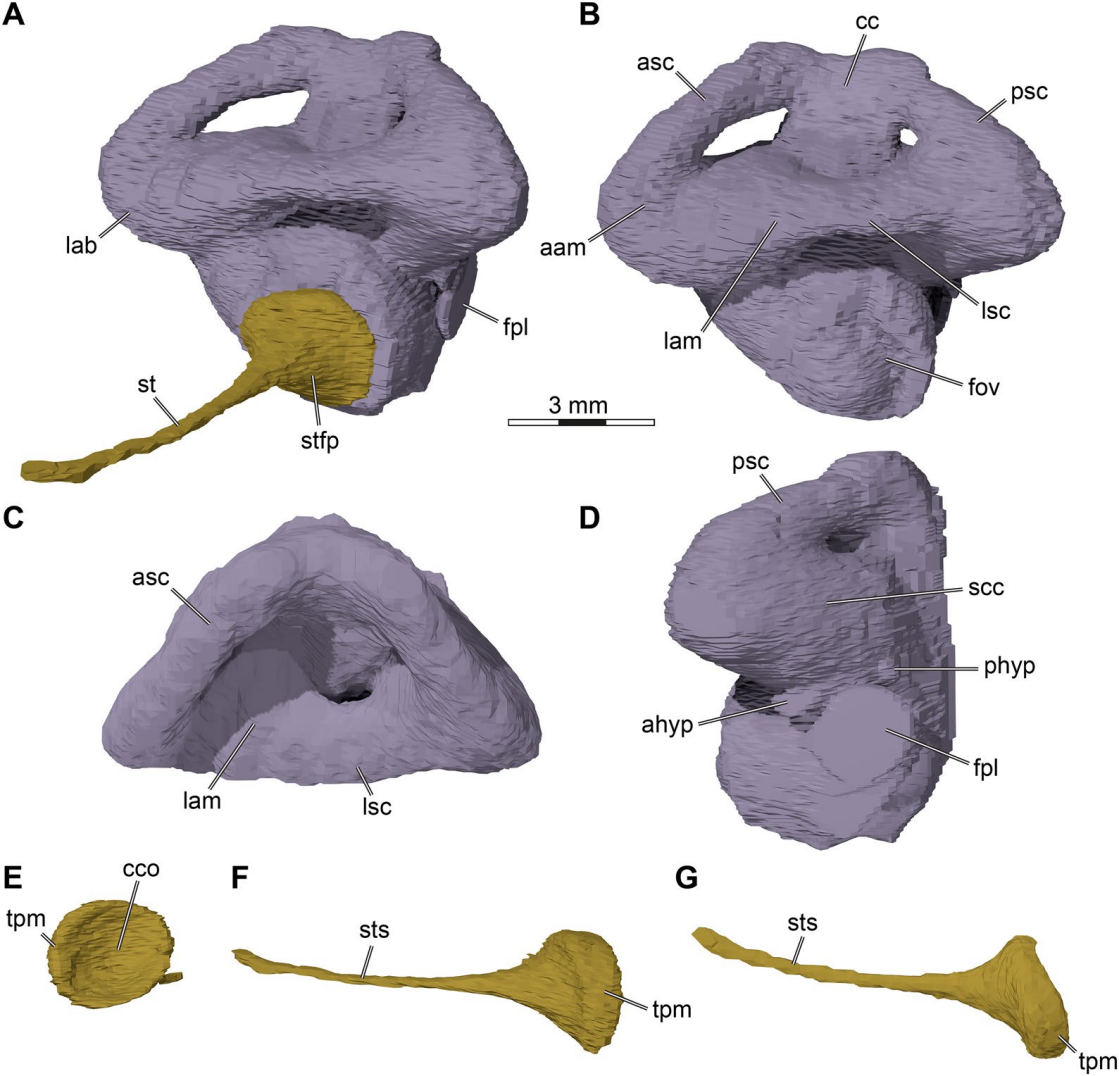
3D renderings of the left endosseous labyrinth and stapes of FMNH UC 1501, *Stylemys nebrascensis*. **A** Labyrinth and stapes in articulation and posterolateral view. **B** Labyrinth in lateral view. **C** Labyrinth in dorsal view. **D** Labyrinth in posterior view. **E** Stapes in medial view, with posterior side to left and dorsal side to top of image. **F** Stapes in posterior view. **G** Stapes in dorsal view. aam, anterior ampulla; ahyp, anterior hypoglossal nerve endocast; asc, anterior semicircular canal; cc, common crus; cco, central concavity of medial side of stapedial footplate; fov, fenestra ovalis; fpl, fenestra perilymphatica; lab, endosseous labyrinth; lam, lateral ampulla; lsc, lateral semicircular canal; phyp, posterior hypoglossal nerve endocast; psc, posterior semicircular canal; scc, secondary common crus; st, stapes; stfp, stapedial footplate; sts, stapedial shaft; tpm, thickened posterior margin of stapedial footplate

The stapes is preserved on the left side in FMNH UC 1501 (Figs. 11B, D, 12A, E, F, G), and it is partially preserved on both sides of UMMP 9318 (Fig. 6C). The right partial stapes has been segmented for the latter specimen. Of the right stapes of UMP 9318, only the stapedial footplate and the medialmost end of the stapedial rod is preserved. The stapedial footplate of *Stylemys nebrascensis* has the common, trumpet shape form for turtles: the footplate is rounded, gently concave on the inner surface facing the labyrinth, and bears a roughly centrally placed, laterally projecting stapedial rod on its lateral surface (Fig. 12E, F, G). The well-preserved stapes of FMNH UC 1501 shows that the margin of the stapedial footplate has varying thickness, with the posterodorsal margin being notably thickened with respect to the remainder of the near-circular margin (Fig. 12E, G). The stapedial rod is quite thin and gracile (Fig. 12A, F, G), possibly explaining why it has been broken so close to the stapedial footplate in UMMP 9318. FMNH UC 1501 shows that the stapedial rod is slightly curved along its course, with the lateral part being slightly posteriorly bend with respect to the central pieces of the rod (Fig. 12G).

### Endosseous labyrinth

We segmented the endosseous labyrinth of the left side of FMNH UC 1501, as the stapes is relatively complete on the left side of this specimen and still in articulation with the fenestra ovalis (Fig. 12A). The labyrinth organ of *Stylemys nebrascensis* is housed in a series of canals and cavities that are formed by the prootic, opisthotic and supraoccipital, as in most turtles (e.g., Ferreira et al., 2022; Gaffney, 1979). The labyrinth has a roughly symmetrical, pyramidal shape in lateral view that is seen across all turtles, in which the vertical semicircular canals have roughly equal lengths (Evers et al., 2022). The semicircular canals of *Stylemys nebrascensis* are thick, such that the spaces between the saccular area and the canals is small, particularly for the lateral and posterior semicircular canal (Fig. 12B–D). Thick semicircular canals haven been mentioned for testudinids before (Evers et al., 2019b). The common crus of *Stylemys nebrascensis* is only very weakly embayed (Fig. 12B). Posterior and lateral semicircular canals share parts of their course in a single cavity, the secondary common crus, such that the posterior ampulla is completely indistinct in the endosseous labyrinth model (Fig. 12D). The lateral ampulla is evident as a major swollen area toward the anterior end of the lateral semicircular canal, and it merges laterally with the region of the anterior ampulla. The lateral semicircular canal is barely laterally curved away from the saccule (Fig. 12C). On the posterior side of the labyrinth, the hypoglossal nerve endocast was included into the model, as this nerve passes through the labyrinth spaces (Fig. 12D). The fenestra perilymphatica opens on the posterior side of the inner ear, and ventral to the upper labyrinth division that is composed of the semicircular canals.

### Mandible

**Fig. 13:**
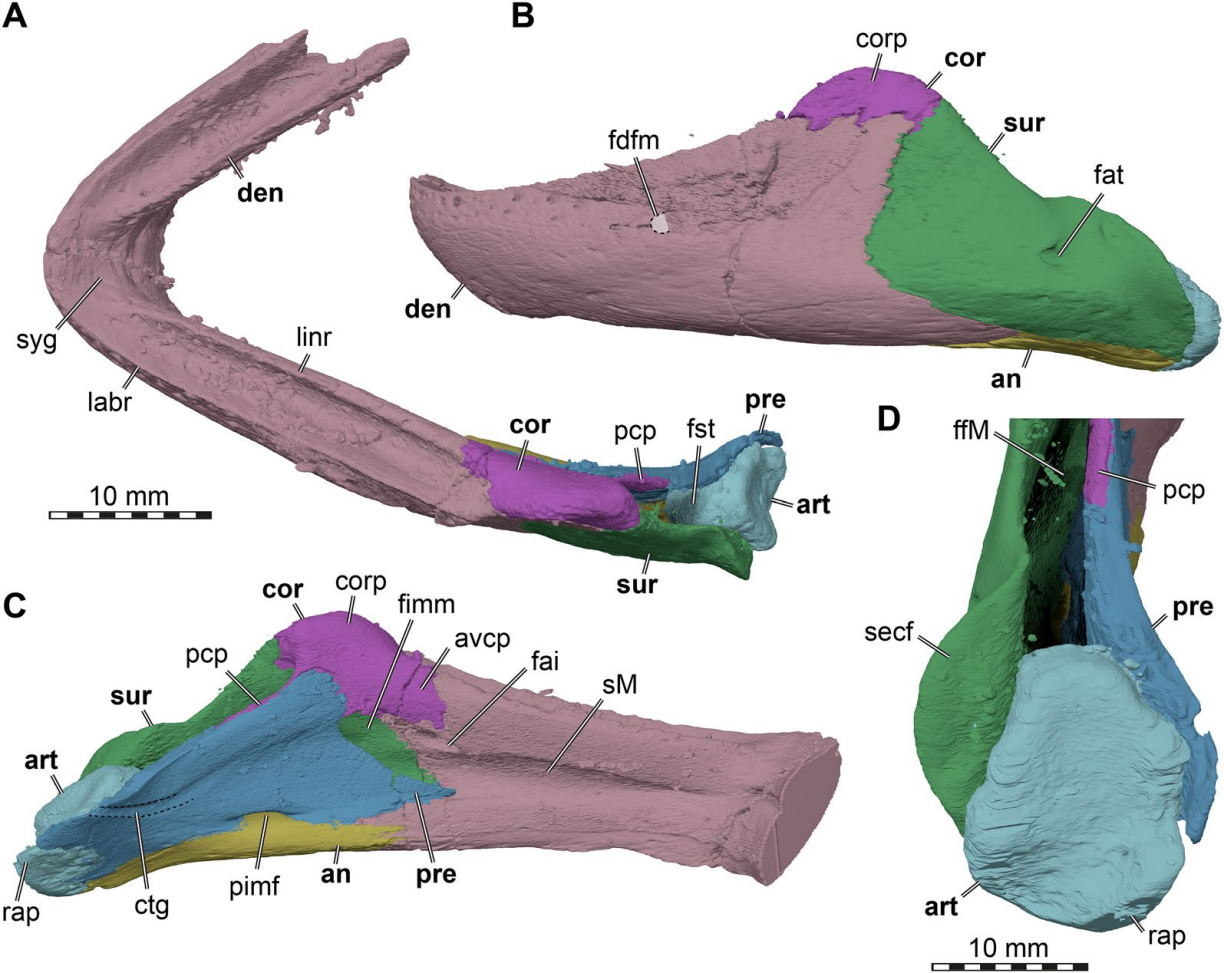
3D renderings of the mandible of FMNH UC 1501, referred to *Stylemys nebrascensis.* **A** Mandible as preserved in dorsal view. **B** Lateral view of left mandibular ramus. **C** Medial view of left mandibular ramus. **D** Close-up onto articulation area of left mandibular ramus. Note that bones are labelled in bold font, and traits in regular font. Abbreviations: an, angular; art, articular; avcp, anteroventral coronoid process; cor, coronoid; corp, coronoid process; ctg, chorda tympani groove; den, dentary; fai, foramen alveolare inferius; fat, foramen auriculotemporalis; fdfm, foramen dentofaciale majus; fim, foramen intermandibulare medius; ffM, foramen into fossa Meckelii; labr, labial ridge; linr, lingual ridge; pcp, posteromedial coronoid process; pimf, posterior intermandibular foramen; pre, prearticular; rap, retroarticular process; secf, surangular ectocondylar flange; sM, sulcus Meckelii; sur, surangular; syg, symphyseal groove

**Fig. 14:**
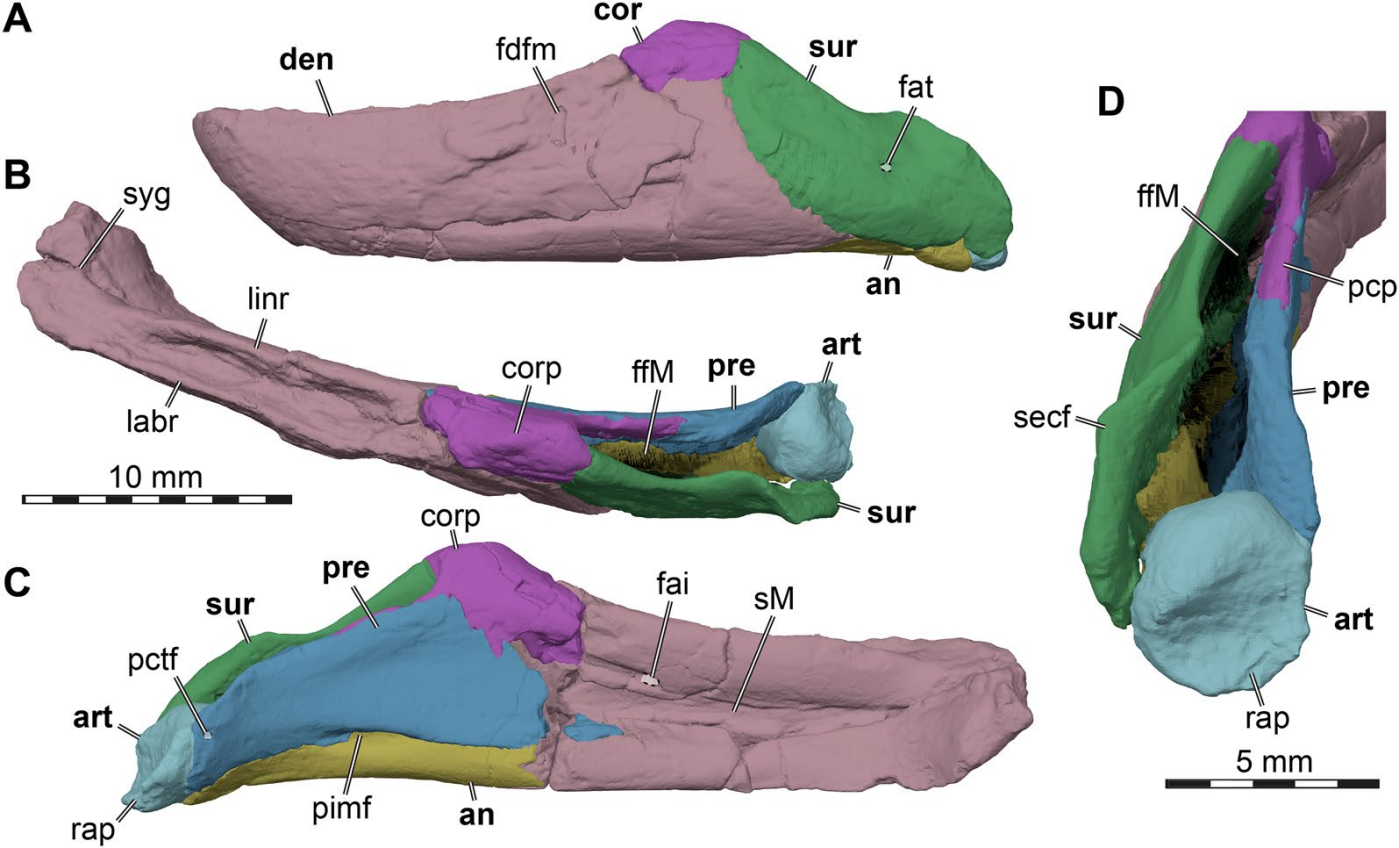
3D renderings of the left mandibular ramus of UMMP 9318, referred to *Stylemys nebrascensis.* **A** Lateral view. **B** Dorsal view. **C** Medial view. **D** Close-up onto articulation area. Note that bones are labelled in bold font, and traits in regular font. Abbreviations: an, angular; art, articular; cor, coronoid; corp, coronoid process; den, dentary; fai, foramen alveolare inferius; fat, foramen auriculotemporalis; fdfm, foramen dentofaciale majus; fim, foramen intermandibulare medius; fM, foramen into fossa Meckelii; labr, labial ridge; linr, lingual ridge; pcp, posteromedial coronoid process; pctf, posterior chorda tympani foramen; pimf, posterior intermandibular foramen; pre, prearticular; rap, retroarticular process; secf, surangular ectocondylar flange; sM, sulcus Meckelii; sur, surangular; syg, symphyseal groove

The mandible of FMNH UC 1501 is only partially preserved, as the posterior part of the right mandibular ramus is not preserved (Fig. 13). However, as the complete left ramus including the symphyseal area are preserved, the full anatomy of *Stylemys nebrascensis* can be described based on FMNH UC 1501. In addition, the mandible of UMMP 9318 is also preserved, although it is generally more fractured than FMNH UC 1501, especially in the symphyseal area (Fig. 14).

### Dentary

The dentary is fully preserved on the left side, and partially on the right side in FMNH UC 1501 (Fig. 13A–C). In UMMP 9318, both dentaries are preserved, but fractured along the symphyseal area and the lateral surface (Fig. 14A–C). The dentaries of *Stylemys nebrascensis* are fully fused, as in most turtles (Evers et al., 2023; Gaffney, 1979). The dentary contacts the coronoid posterodorsally, the surangular posteriorly, the angular posteroventrally, and the prearticular on the medial jaw surface along the anteroventral process of the latter.

**Fig. 15:**
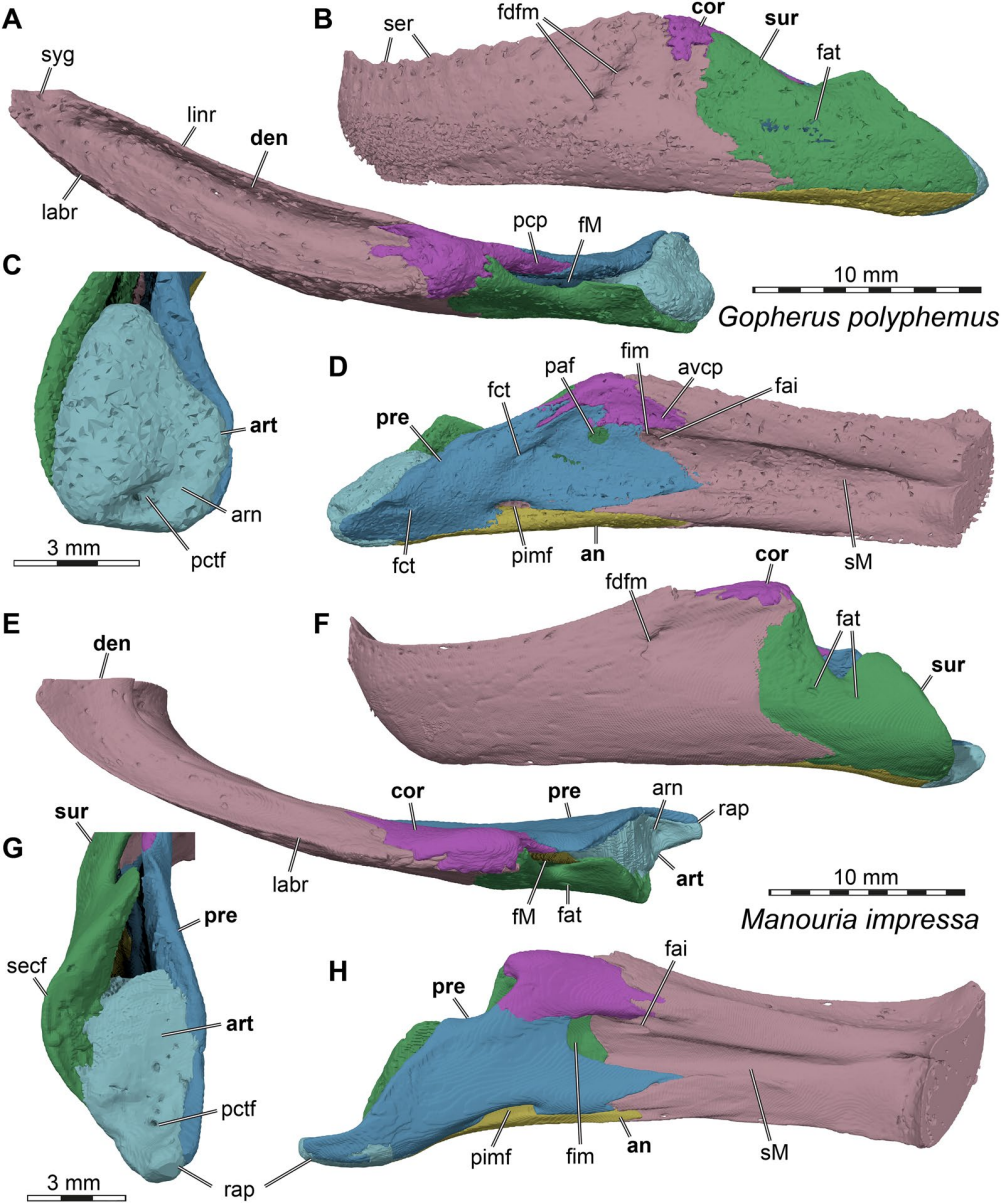
3D renderings of the left mandibular rami of *Gopherus polyphemus* and *Manouria impressa.* **A**–**D** *Gopherus polyphemus* (FMNH 211815). **A** Dorsal view. **B** Lateral view. **C** Close-up of articulation area. **D** Medial view. **E**–**H** *Manouria impressa* (SMF 69777). **E** Dorsal view. **F** Lateral view. **G** Close-up of articulation area. **H** Medial view. Note that bones are labelled in bold font, and traits in regular font. an, angular; arn, articular notch; art, articular; avcp, anteroventral coronoid process; cor, coronoid; den, dentary; fai, foramen alveolare inferius; fat, foramen auriculotemporalis; fct, foramen chorda tympani; fdfm, foramen dentofaciale majus; fim, foramen intermandibulare medius; fM, foramen into fossa Meckelii; labr, labial ridge; linr, lingual ridge; paf, prearticular foramen; pcp, posteromedial coronoid process; pctf, posterior chorda tympani foramen; pimf, posterior intermandibular foramen; pre, prearticular; rap, retroarticular process; secf, surangular ectocondylar flange; ser, serrations; sM, sulcus Meckelii; sur, surangular; syg, symphyseal groove

As in other testudinids (Evers et al., 2023), the dentary is relatively straight, with most of the curvature limited to the symphyseal part of the dentary. The dentary is dorsoventrally deep, and the symphysis lacks an upcurved hook or symphyseal ridge (Figs. 13, 14). The dentary has deep labial and lingual ridges (Figs. 13A, 14B). Anteriorly, the lingual ridge becomes gradually weaker, creating a symphyseal gap in the ridge that discontinues the narrow but deep trough between the triturating ridges (Figs. 13A, 14B). The triturating ridges are mildly serrated in FMNH UC 1501, as in many testudinids (Evers et al., 2023). Serrations cannot be confirmed in UMMP 9318, but the lingual margin is also difficultly preserved so that the scans are not entirely conclusive for this feature. The triturating surface morphology of *Stylemys nebrascensis* is nearly identical to that of *Gopherus polyphemus* (Evers et al., 2023; Fig. 15A), but it differs distinctly from that of *Manouria impressa*, in that the latter lacks a protruding lingual ridge over most of its lingual dentary margin (Fig. 15E).

A processus dentofacialis is absent in *Stylemys nebrascensis*, just like in other testudinids (Evers et al., 2023). The foramen dentofaciale majus is positioned on the lateral dentary surface and is small (Figs. 13B, 14A) as in testudinids generally (Evers et al., 2023), but even smaller than the one in *Gopherus polyphemus*.

Medially, the dentary surface of *Stylemys nebrascensis* is dominated by a large but shallow Meckellian groove (Figs. 13C, 14C). It is less distinct than in *Manouria impressa* and *Gopherus polyphemus* (Fig. 15D, H)*.* The foramen alveolare inferius is located in the medial surface of the dentary, and its position is not concealed by the coronoid or prearticular (Figs. 13C, 14C).

### Surangular

The surangular is only preserved on the left side of the FMNH UC 1501, but on both sides of UMMP 9318. The surangular of *Stylemys nebrascensis* contacts the coronoid anterodorsally, the dentary anteriorly, the angular ventrally, and the articular posteriorly (Figs. 13, 14). A contact with the prearticular is also present, albeit only in the anterior parts of the floor of the fossa Meckelii, which is not apparent from lateral or medial views alone and thus difficult to observed without digital dissection of the mandible.

The surangular of *Stylemys nebrascensis* has a large lateral exposure on the jaw (Figs. 13B, 14A), as is generally the case in testudinids (Evers et al., 2023). It lacks the anterior surangular process that is present in testudinines, and this is consistent with turtles from the *Gopherus* + *Manouria* clade (Fig. 15B, F). The dorsal process of the surangular of *Stylemys nebrascensis* is in contact with the coronoid, and it forms the lateral margin of the fossa Meckelii. The dorsal foramen into the fossa Meckelii is a broad opening (Figs. 13A, D, 14B, D) that is unlike the narrower, more constricted openings seen in *Gopherus polyphemus* and *Manouria impressa* (Fig. 15A, E). Posteriorly, the foramen into the fossa Meckelii is open toward the articular, meaning there is no posterodorsal contact of the surangular with the prearticular (Figs. 13D, 14D), which is in agreement with *Gopherus polyphemus* and *Manouria impressa* (Fig. 15C, G). As in testudinids generally (Evers et al., 2023), *Stylemys nebrascensis* lacks a medially recurved surangular lamina that would project into the fossa Meckelii. The foramen auriculotemporalis is relatively small (Figs. 13B, 14A), as in other testudinids (Evers et al., 2023), and a dorsal surangular foramen is absent. The lateral surangular flange for the ectocondyle of the quadrate is absent in most testudinids (Evers et al., 2023), however it is present in the *Stylemys nebrascensis* (Figs. 13D, 14D) and in *Manouria impressa* (Fig. 15G) and *Gopherus agassizii*, although our specimen of *Gopherus polyphemus* lacks the structure (Fig. 15C)*.* The posterior margin of the surangular of *Stylemys nebrascensis* thus contributes to the lateral margin of the articular surfaces of the jaw articulation.

### Coronoid

The coronoid is only preserved on the left side of the FMNH UC 1501, but present on both sides of UMMP 9318. It is a small, mount-like bone that forms the coronoid process for the attachment of the coronar aponeuroses tendon (Evers et al., 2023). Despite its relatively low morphology in *Stylemys nebrascensis* (Figs. 13A–C, 14A–C), it is more prominent than in most other testudinids, including gopher tortoises, in which the coronoid barely projects dorsally above the level of the highest point of the dentary (Evers et al., 2023; Fig. 15B). The coronoid of *Stylemys nebrascensis* shows the regular contacts, with the dentary anteriorly, the surangular posterolaterally, and the prearticular medially. The coronoid contributes to the formation of the dorsal margin of the fossa Meckelii by bridging over the surangular and prearticular (Figs. 13A, 14B). The anteroventral process of the coronoid in *Stylemys nebrascensis* is present yet short (Figs. 13C, 14C), and lacks a coronoid foramen. A posteroventral process that extends along the medial margin of the foramen into the fossa Meckelii is present, and it excludes the prearticular from the anterior part of the margin (Figs. 13A, D, 14B, D), as in species of the *Gopherus* + *Manouria* clade (Fig. 15A, E), but unlike in Testudininae (Evers et al., 2023).

### Prearticular

The prearticular is a thin, sheeted bone that is only preserved on the left side of the FMNH UC 1501 but on both sides of UMMP 9318, where the anterior parts of the bone are broken and damaged on both sides. The prearticular of *Stylemys nebrascensis* forms the medial wall of the fossa Meckelii (Figs. 13C, 14C), and contacts the surangular in the floor of this structure. Other contacts are with the coronoid along an anterodorsal process, with the angular along most of the ventral margin, and with the dentary at the anterior end of an anteroventral process. The prearticular diverges anteriorly into a dorsal and ventral process, which form the foramen intermandibulare medius between the fossa Meckelii and the intermandibular space (Figs. 13C, 14C). Hereby, the dorsal and ventral processes are not well separated, but connected by a bony sheet, so that the foramen intermandibulare medius is effectively formed between prearticular and coronoid. A prearticular foramen is absent. This morphology is as in *Manouria impressa* (Fig. 15H) and *Gopherus agassizii* (Evers et al., 2023), but unlike in *Gopherus polyphemus*, in which the prearticular is ossified between the dorsal and ventral rami, also enclosing a prearticular foramen (Evers et al., 2023; Fig. 15D). Together with the angular, the prearticular of *Stylemys nebrascensis* forms a relatively large, singular intermandibular foramen (Figs. 13C, 14C), as is also the case in gopher tortoises (Evers et al., 2023) and *Manouria impressa* (Fig. 15D, H). At its posterior end, the prearticular of *Stylemys nebrascensis* frames the articular medially, hereby forming a small medial flange that participates in the formation of the articulation surface of the mandible (Figs. 13D, 14D). UMMP 9318 and FMNH UC 1501 both show signs of a medially placed chorda tympani nerve course, although they slightly differ in their morphology. In FMNH UC 1501, the medial surface of the prearticular is incised by a fine groove, which extends from the margin of the articulation area anteroventrally across the bone before it vanishes (Fig. 13C). Thus, the nerve course is not internalized to a canal in this specimen. In UMMP 9318, there is a small foramen instead near the articulation margin pf the prearticular (Fig. 14C), and a thin canal extends from there forward and into the fossa Meckelii. Despite the described variation, it seems that *Stylemys nebrascensis* therefore has a chorda tympani nerve course via the prearticular, and not via the articular bone. This differs from *Gopherus polyphemus* or *Gopherus agassizii* (Evers et al., 2023; Fig. 15C) insofar as these species seem to have two chorda tympani nerve courses (one through the articular, and one groove along the prearticular), but also from the condition in our specimen of *Manouria impressa*, which lacks a medial chorda tympani canal or foramen and instead only has a respective foramen in the articular bone (Fig. 15G).

### Angular

The angular is only preserved fully on the left side of the FMNH UC 1501, but completely preserved on both sides of UMMP 9318. It is a thin and elongate bone at the ventral margin of the posterior jaw end that forms a ventral cap between the prearticular and surangular (Figs. 13C, 14C), thereby forming the floor of the posterior part of the fossa Meckelii. Its posterior end buttresses the articular from ventral. The angular contributes to the formation of the ventral margin of the posterior intermandibular foramen (Figs. 13C, 14C). Anteriorly, the angular contacts to the dentary.

### Articular

The articular is a small chunky bone, only preserved fully on the left side of the FMNH UC 1501, but on both sides of UMMP 9318. It is posteriorly wedged between the angular, prearticular and surangular and forms almost the entire surface of the articular surface of the jaw that faces the quadrate (Figs. 13D, 14D). The dorsal surface is largely concave, but slightly raised toward the contact with the surangular, which results in a low, vertical ridge that effectively separates the articulation area into a medial and lateral subfacet (Figs. 13D, 14D), as is common in cryptodires (Evers et al., 2023). The articular of *Stylemys nebrascensis* also forms a small retroarticular process, which is developed as a small, dorsally upcurved lip at the posteromedial end of the bone (Figs. 13D, 14D). The retroarticular process is separated from the articular surface by a small grooved posterior notch that is obliquely oriented. The morphology of the articular notch and retroarticular process of FMNH UC 1501 closely agrees with that seen in gopher tortoises (Evers et al., 2023; Fig. 15C), whereas *Manouria impressa* shows a longer and more recurved, hook-like retroarticular process (Fig. 15E, G, H).

## Discussion

### *Taxonomic notes on* Stylemys nebrascensis

In his recent taxonomic revision of North American testudinoids, Vlachos (2018) retains three valid species of the genus *Stylemys*, and lays out diagnoses for all three, including the type species, *Stylemys nebrascensis*. These form the basis for referring the principal specimens under study here to *Stylemys nebrascensis* (see section Referral of both specimens to *Stylemys nebrascensis*, above). There is some evidence that indeed uphold small morphological variations between the three holotypes of the nominal species of *Stylemys* following Vlachos (2018), which include the posterior gular extent with regard to the entoplastron and the relative lengths of the femoral and anal scute midlines (Vlachos, 2018). These latter two traits could be used in a differential diagnosis for *Stylemys nebrascensis*, and we use them to exclude the possibility that UMMP 9318 may belong to one of the other nominal species of *Stylemys*. In addition, *Stylemys capax* is geographically separated from *Stylemys nebrascensis* (Vlachos, 2018), whereas *Stylemys inusitata* is Miocene in age and thus stratigraphically younger than the other two species.

Our own observations and a synthesis of observations from previous studies (e.g., Auffenberg, 1976; Hay, 1908; Hutchison, 1996) allow us to expand the diagnosis for *Stylemys nebrascensis* with regard to the diagnosis provided by Vlachos (2018). As the holotype of *Stylemys nebrascensis* is an incompletely preserved shell (Fig. 1), we highlight a few diagnostic features here that can be derived from the holotype (USNM 97) itself, and which can be used to refer specimens to *Stylemys nebrascensis*: the holotype specimen combines a relatively small size with thick bone and a relatively high-domed carapace. Additionally, the specimen has a neural pattern without posteriorly short sided hexagonal shapes (4 > 6A > 6A > 6A > 6A > 6A > 6A > 6A; Fig. 1A, D), a humero-pectoral sulcus that is laterally strongly curved to the anterior (Fig. 1B, E), a small inguinal scute without femoral contact (Fig. 1B, E), and a pleural I that does not extend onto the peripheral bones (Fig. 1A, D). Although one of the diagnostic features for *Stylemys nebrascensis* is that its humero-pectoral sulcus is posteriorly positioned with regard to the entoplastron, the holotype specimen USMN 97 actually shows a contact between entoplastron and this scute sulcus (Fig. 1B, E)—but the sulcus does not extend onto the entoplastron, as it does, for instance, in “*Testudo*” *brontops* (Hutchison, 1996: Fig. 7). This nicely highlights that many features, including diagnostic features, are subject to variation. Nevertheless, the combination of the above features allows to confirm the historic referral of many addition specimens to *Stylemys nebrascensis*, including UMMP 9318 (Case, 1925), thus broadening the pool of more complete specimens from which further diagnostic characters can be derived. On the basis of referred material that can be safely attributed to the species, we synthesized a diagnosis that includes characters which are agreed upon between various authors who have dedicated time to studying *Stylemys nebrascensis* (e.g., Auffenberg, 1964; Hay, 1908; Hutchison, 1996; Vlachos, 2018) and which is expanded by additional skull characters that have previously not been noted. However, we limit our diagnostic traits to the shell and skull, as a postcranial revision is outstanding. In addition, our diagnosis provides statements that differentiate *Stylemys nebrascensis* from other fossil turtles that are found in the White River Group (e.g., *Oligopherus laticuneus*; “*Testudo*” *brontops*; Hutchison, 1996). This is an important addition to the diagnoses given by Vlachos (2018), which included some characters that are present in multiple co-occurring species (e.g., interpremaxillary ridge in *Stylemys nebrascensis* and *Oligopherus laticuneus*).

Our revised differential diagnosis recognizes features that can, in principle, be subject to ontogenetic or sexually dimorphic differences (e.g., Angielczyk & Feldman, 2013; Fish & Stayton, 2014; Smith et al., 2001). This particularly concerns the differences in size, bone thickness, shell doming, and the size of the gular protrusions on the anterior plastral lobe between *Stylemys* spp. and *Oligopherus laticuneus*. Evidence that these features indeed reflect taxonomy comes from known sexual differences in the shapes of the gular protrusion for *Oligopherus laticuneus* (Hutchison, 1996), and the list of morphological features that vary between *Stylemys* spp. and *Oligopherus laticuneus* which can unlikely be explained by ontogenetic change (e.g., differences in neural formulae and various differences in the cranium).

One of the key features in distinguishing *Stylemys* spp. from *Oligopherus laticuneus* – differences in their neural formula (e.g., Gilmore, 1946; Hutchison, 1996) – are subject to intraspecific variation. For example, while the holotype of *Stylemys nebrascensis* (USNM 97; Fig. 1A, D) and the holotype of *Stylemys inusitata* (CM 311; Hay, 1908: plate 68) have a neural pattern of 4 > 6A > 6A > 6A > 6A > 6A > 6A > 6A, the holotype of *Stylemys lata* (now synonymized with *Stylemys nebrascensis*; Vlachos, 2018; Auffenberg, 1964: Fig. 5) and the holotype of *Stylemys capax* (AMNH 1357; Hay, 1908: Fig. 499) have a neural pattern of 4 > 8 > 4 > 6A > 6A > 6A > 6 > 6 > 6. Variation in the neural patterns is also evident from other Oligocene tortoises from North America. For instance, the holotype of *Oligopherus laticuneus* (holotype of *Testudo laticunea*) has a neural pattern (AMNH 1160: 6P > 6P > 4 > 6A > 4 > 6 > 6A > 8; Hay, 1908: Fig. 509) that is clearly distinct from that of *Stylemys nebrascensis* (and other nominal *Stylemys* species) by the presence of posteriorly short sided hexagonal neurals and the presence of rectangular neurals in positions that differ from those of *Stylemys* spp. However, various specimens that have been referred to *Oligopherus laticuneus* and which in part are holotypes of subjective junior synonyms of this species vary from the pattern seen in the holotype. Two important specimens in this regard are the holotype of *Testudo praeextans* (CNM 840: 4 > 6P > 4 > 6A > 6A > 6A > 6A > 6A; Gilmore, 1946: Fig. 24) or USNM 15874 (4 > 8 > 4 > 8 > 6A > 6A > 6 > 6A; Gilmore, 1946: plate 38). The latter specimen is important because it consists of a basically complete shell, a cranium and a mandible and is thus used as an exemplar specimen of *Oligopherus laticuneus* (e.g., Vlachos, 2018), for example for phylogenetic characterizations of the species (e.g., Vlachos & Rabi, 2018). Overall, neither *Oligopherus laticuneus* nor *Stylemys nebrascensis* have consistent neural shape patterns even when considering the few specimens mentioned here. These variations in *Stylemys nebrascensis* and *Oligopherus laticuneus* have in the past been interpreted as an indication of a poor diagnostic value of neural formulae at least for Oligocene tortoises (e.g., Auffenberg, 1964; Hay, 1908). Nevertheless, within a range of variation, there seem to be differences at least on the genus level between *Stylemys* and *Oligopherus*. In our revised diagnosis of shell characteristics, we therefore modified the ‘definition’ of the neural pattern for *Stylemys* to a include aspects of variation that seems to occur in the species. Specifically, we propose that *Stylemys* spp. can be identified and differentiated from other temporally and spatially co-occurring fossil tortoises based on a neural pattern in which no hexagonal neurals with posteriorly short sides (i.e. 6P shapes) are present and in which at most one octagonal neural exists, in the second neural position. This contrasts with specimens of *Oligopherus laticuneus*, in which either 6P neurals or several octagonal neurals occur. High amounts of neural pattern variation among tortoises have also been noted for the extant *Gopherus* spp. (e.g., Auffenberg, 1976; Pritchard, 1988). As such, the relatively frequent variations in the fossils *Stylemys nebrascensis* and *Oligopherus laticuneus* may support hypotheses about their affinities with the *Gopherus*-lineage.

The presence of a premaxillae ridge with a corresponding symphyseal groove in the mandible is historically important in recognizing *Stylemys nebrascensis* (Hay, 1908; Case, 1925, Auffenberg, 1984; Crumly, 1994; Vlachos, 2018). Given that *Stylemys inusitata* and *Stylemys capax* lack cranial material, the ridge could equally be diagnostic on the genus level (Vlachos, 2018). However, a premaxillary ridge is also present in the material referred to *Testudo praeextans* (Gilmore, 1946; Lambe, 1913; Vlachos, 2018; USNM 15874), which has since been synonymized with *Oligopherus laticuneus* (Vlachos, 2018). As *Oligopherus laticuneus* and *Stylemys nebrascensis* have overlapping temporal and geographic ranges (Hutchison, 1996; Vlachos, 2018), the mere presence of a premaxillary ridge is not a particularly useful diagnostic feature for either of these species. However, our anatomical work shows that the premaxillary ridge of the *Stylemys nebrascensis* specimen for which this feature is preserved (FMNH UC 1501) is low and relatively robust, contrasting the thin and sharp-edged ridges of *Gopherus* spp. and *Oligopherus laticuneus* (Gilmore, 1946: Fig. 22). As we have not examined the *Oligopherus laticuneus* material (USNM 15874) ourselves, we cautiously suggest that there may be a diagnostic difference to the robustness of the ridge between *Stylemys nebrascensis* and *Oligopherus laticuneus*.

### The value of detailed anatomical skull descriptions

Our detailed anatomical descriptions of the cranium and mandible of specimens we refer to *Stylemys nebrascensis* reveal not only potential diagnostic characteristics for the species based on the skull, but also show that testudinid skulls are not well characterized in the current literature landscape. Several features, such as the poststapedial process of *Stylemys nebrascensis*, are herein described for the first time, although we found them in extant tortoises used for comparisons. These features have a high potential for future phylogenetic studies, as they represent previously undocumented variation and similarities between fossil and extant specimens that may help elucidate tortoise phylogeny and morphological evolution.

### Comments on the cranial anatomy and evolution of *Stylemys nebrascensis*

Our anatomical work reveals many features for *Stylemys nebrascensis* that are currently not known from other testudinids. However, other fossil North American tortoise skulls, particularly *Oligopherus laticuneus* but also *Hesperotestudo* spp. are currently not described or figured well enough to make detailed comparisons. It is clear that these specimens require anatomical descriptions, but these were beyond the scope of this study. Nevertheless, we provide detailed comparative descriptive observations for the extant tortoises *Gopherus polyphemus* and *Manouria impressa*, which presumably bracket *Stylemys nebrascensis* and at least some other fossil testudinids from North America. In the following, we list features that are present in *Stylemys nebrascensis*, but not in *Gopherus polyphemus* or *Manouria impressa*. We consider them as potentially diagnostic for *Stylemys nebrascensis*, with the caveat that other North American fossil tortoises, particularly *Oligopherus laticuneus*, *Hesperotestudo* spp., and the other valid *Stylemys* species are not well described, making it currently impossible to fully access if some of the features listed below may be more widespread than currently recognized. For some features, we tentatively comment on the morphology of *Oligopherus laticuneus* based on the figures provided in Gilmore (1946). As our list is based on observations of two specimens (FMNH UC 1501, UMMP 9318), we also indicate which of the features can be confirmed to be identical in both specimens.

Unique features of *Stylemys nebrascensis* under the comparative framework of this study are: the presence of a medial process of the jugal with resulting jugal contacts with the palatine and pterygoid (present in both specimens, absent in all extant tortoises, plesiomorphically present in testudinoids; absent in *Oligopherus laticuneus* based on dorsally visible jugal sutures in Gilmore, 1946: Fig. 20); a lingual ridge of the maxilla that is only low (present in both specimens, present and deep in *Gopherus* spp., absent in *Manouria impressa*); ventral vomer keel with robust morphology (present in both specimens, keel is thin in *Gopherus* spp., and low and anteriorly restricted in *Manouria impressa*); small epipterygoid size that is restricted to an area ventral to the subtrigeminal ridge of the pterygoid (present in both specimens, epipterygoids large in *Gopherus* spp. and *Manouria impressa*); presence of an epipterygoid–quadrate contact (only confirmed in FMNH UC 1501, as damaged in UMMP 9318, contact is absent in *Gopherus polyphemus* and *Manouria impressa*); processus trochlearis oticum formed as a protruding, circular peduncle with a central, circular depression (present in both specimens; processus is convex and not concave in *Gopherus* spp. and *Manouria impressa*); a short articular process of the quadrate that does not project ventrally far beyond the margin of the cavum tympani (present in both specimens, process projects ventrally deeply beyond cavum tympani in *Gopherus* spp. and *Manouria impressa*); presence of a subcavernosal foramen and canal between quadrate and pterygoid (present in both specimens, absent in all other testudinids examined; not indicated in drawing of *Oligopherus laticuneus* in Gilmore, 1946: Fig. 22); pterygoid fossa somewhat deeper developed than in comparative extant species, but still shallow (present in both specimens; pterygoid area between quadrate and parabasisphenoid nearly completely flat in *Gopherus* spp. and *Manouria impressa*); foramen posterius canalis carotici interni (fpcci) completely enclosed by the pterygoid directly ventral to the margin of the fenestra postotica (observed in FMNH UC 1501, inferred for UMMP 9318 which is partially damaged in this region; fpcci begins as dorsally open groove in the cavum acustico-jugulare in other testudinids); presence of a poststapedial process of the pterygoid that makes contact with the opisthotic within the cavum acustico-jugulare, thereby forming a poststapedial foramen between the fenestra postotic region and the lateral part of the recessus scalae tympani (confirmed only in FMNH UC 1501, as the area is broken in UMMP 9318; absent in *Gopherus* spp. and *Manouria impressa*, but present in some Testudininae); anterior foramen for the vidian nerve exits the skull on the lateral surface of the pterygoid slightly anterior to the epipterygoid position (present in both specimens; vidian canal extends through the pterygoid and into the palatine in *Gopherus polyphemus* and *Manouria impressa*, from which it exits in a comparatively further anterior position between the anterior end of the secondary lateral braincase wall and the foramen palatinum posterius); opisthotic–squamosal contact along the paroccipital process is narrowly absent (confirmed only in UMMP 9318, as the area is broken in FMNH UC 1501; contact is present in *Gopherus polyphemus* and *Manouria impressa*); broad, large dorsal foramen into the fossa Meckelii in mandible (present in both specimens; the opening is transversely narrowly constricted in *Gopherus polyphemus* and *Manouria impressa*).

We also observed similarities between *Stylemys nebrascensis* and *Manouria impressa* to the exclusion of *Gopherus polyphemus*. These may be diagnostic of *Stylemys nebrascensis* among North American tortoises. Evolutionarily, these could represent symplesiomorphies if *Stylemys nebrascensis* is closer related to *Gopherus* spp. than to *Manouria* spp., which we believe is a reasonable hypothesis given the shared presence of some characters in *Stylemys nebrascensis* and gopher tortoises that are unknown from taxa outside of North America, such as the interpremaxillary ridge. It should again be said that the following features were not examined for other fossil North American tortoises, and may thus have a wider distribution than currently recognized. These features are: the absence of an epipterygoid–palatine contact (present in *Gopherus polyphemus*, but also absent in *Manouria impressa*); full integration of the epipterygoid into the wall of the secondary braincase (absent in *Gopherus polyphemus*, in which the epipterygoid lies only superficially on the lateral braincase wall, but also present in *Manouria impressa*); absence of a separation of the foramen nervi trigemini and the mandibular artery foramen (present in *Gopherus* spp., but also absent in *Manouria impressa*); absence of retractor bulbi pits on the parabasisphenoid (present in *Gopherus polyphemus*, but also absent in *Manouria impressa*); presence of a lateral flange on the external pterygoid process (absent in *Gopherus polyphemus*, but also present in *Manouria impressa*); reduced prootic exposure in the floor of the upper temporal fossa (large in *Gopherus* spp., but also small in *Manouria impressa*).

### Palaeoecological comments on *Stylemys nebrascensis*

Extant gopher tortoises feed predominantly on coarse and tough plant material that grows in semiarid and arid environments (e.g., Bramble, 1974; Ernst & Barbour, 1989). These tortoises are adapted to such a diet with several morphological innovations related to the triturating surfaces and jaw movement. This includes a tongue-in-groove arrangement of the mandibular articulation surface with a pronounced, centrally placed and anteroposteriorly trending ridge and a central sulcus on the corresponding articular process of the quadrate (Fig. 7E), which results in a kinetic adduction system in which the mandible shears from anterior-to-posterior along the upper jaw during the mastication cycle (Bramble, 1971). The median premaxillary ridge and corresponding mandibular symphyseal grove hereby act as a guiding system to prevent the mandible from lateral dislocation (Bramble, 1971), and the accessory triturating ridge of the maxilla and high lingual and labial ridges on the upper and lower jaw result in an effective processing of the plant matter (Bramble, 1971). Although some protraction and retraction movements of the mandible against the upper jaw are generally common in herbivorous turtles (Bramble, 1974), this type of movement is extreme in *Gopherus* spp., and may have facilitated the ossification of the os transiliens as a true sesamoid bone from the transiliens cartilage that is generally observed in turtles (Bramble, 1974). Functionally, the os transiliens brings the adductor musculature into a more vertical line of action, hereby maximizing grinding efficiency (Bramble, 1974). A relatively far anteriorly placed and low coronoid process of the mandible, and a relatively deep mandibular ramus are further mechanical adaptations to herbivory (Bramble, 1971).

*Stylemys nebrascensis* shows several of the adaptations seen in *Gopherus* spp., especially with regard to the triturating morphology. Unless this represents a functional convergence, the morphology of the triturating morphology of *Stylemys nebrascensis* may thus be informative to understand the evolution of this complex in North American tortoises. Specifically, *Stylemys nebrascensis* bears a well-developed, sharp-edged accessory triturating ridge alongside well-developed labial and lingual ridges on the upper jaw surface. These are complemented on the mandible by a dentary triturating surface with a central trough bordered by lingual and labial ridges. These ridges are likely indicative of feeding on relatively tough plant material, as soft-vegetation feeding turtles commonly lack the accessory ridge and have poorly ridged triturating surfaces (Bramble, 1971). In addition, *Stylemys nebrascensis* has a median premaxillary ridge that is opposed by a symphyseal groove on the mandible. Although the median premaxillary ridge (and also the lingual ridge) of *Stylemys nebrascensis* is less deep and less sharp than those of *Gopherus* spp., the function of the ridge in *Gopherus* spp. according to Bramble (1974) suggests that *Stylemys nebrascensis* may already have had a pronounced kinetic adduction mechanism that is typical for *Gopherus* spp. (Bramble, 1971). This may explain the potential presence of os transiliens sesamoids that we hypothesize for *Stylemys nebrascensis*, contra Bramble (1974), based on the presence of circular depressions on the anterodorsal side of the processus trochlearis oticum. However, given that the mandibular articulation of *Stylemys nebrascensis* is less deeply notched than in *Gopherus*, it appears that the sliding mechanism for the jaw was less developed than in the modern species. Overall, the osteology of the feeding apparatus of *Stylemys nebrascensis* indicates a similar diet on hard shrubs and grasses than is observed for extant gopher tortoises, and this is consistent with the spread of open-habitat grasslands across the Great Plains during the late Oligocene and early Miocene (e.g., Strömberg, 2004).

### Increased semicircular canal thickness in testudinids

*Stylemys nebrascensis* is the second tortoise for which thickened semicircular canals have been explicitly reported (see also *Indotestudo elongata* in Evers et al., 2019b), but available 3D models of the endosseous labyrinths of further tortoises (Evers et al., 2022) indicate that tortoises more widely have thick semicircular canals. Thick semicircular canals have otherwise been largely reported for marine turtles (Evers et al., 2019b) as well as other marine lineages, where evolutionary increases in canal thickness have often been interpreted as a diving adaptation in reptiles (e.g., Neenan et al., 2017; Schwab et al., 2020). Given the presence of thick semicircular canals in tortoises, the thickness cannot be explained exclusively as a diving adaptation. Increases in semicircular duct cross-sectional area result in increased response time to rotational accelerations (Georgi & Sipla, 2008; Rabbitt et al., 2004), which may be advantageous in an under-water setting. However, this may also be important for terrestrial tortoises because of their feeding strategy, which involves complex neck-head coordination and which is distinct from the aquatic ingestion of food performed by nearly all other (i.e. non-testudinid) turtles (Bels et al., 2007; Bramble & Wake, 1985; Lemell et al., 2019; Natchev et al., 2015). The correspondence of sizes of the semicircular ducts and their surrounding endosseous canals is possibly not a very direct one for turtles, as has been shown for marine turtles with increased canal thickness (Evers et al., 2022). Thus, it should be tested if the duct sizes of tortoises increase according to their increased canal sizes to assess if terrestrial feeding may explain the observed canal thickness.

### Future directions

The relatively recent study of North American tortoises by Vlachos (2018) is a comprehensive nomenclatural revision of a complex landscape of historic taxa, but it provides few anatomical comments or revisions of previous anatomical observations. However, a robust taxonomy of testudinids also requires detailed anatomical studies of the available materials, and especially of holotypes or specimens that have historically been used to characterize species (such as UMMP 9318 for *Stylemys nebrascensis*). This present work can only but be a first step toward a more systematic re-assessment of fossil North American tortoises. Important specimens to study in the future include USNM 15874 as a particularly complete specimen of presumably *Oligopherus laticuneus*, and various specimens of *Hesperotestudo* spp. and *Hadrianus* spp. Future skull descriptions of these taxa will allow for better phylogenies of tortoises, which provide the framework for understanding many aspects of evolution, including the morphological evolution of the specialized ecology of gopher tortoises.

## Conclusions

Although *Stylemys nebrascensis* is a long-known fossil tortoise, most aspects of its anatomy are poorly resolved. Our work provides anatomical and taxonomic comments on the shell morphology that the species is originally based on, as well as the detailed cranial and mandibular osteology of referred specimens. *Stylemys nebrascensis* shares many anatomical features with extant gopher tortoises, which lends support to the hypothesis that all North American fossil tortoises may be related to modern gopher tortoises, forming a geographically localized clade that split from related Asian tortoises sometime between the Eocene and Oligocene. This hypothesis should be tested through detailed descriptions of further fossil material of North American tortoises, which will allow a comprehensive phylogenetic assessment. *Stylemys nebrascensis* likely was a dietary specialist feeding on tough and fibrous plants, as indicated by anatomical similarities to modern gopher tortoises that include strongly ridged triturating surfaces, a kinetic jaw retraction mechanism, and likely the presence of ossified sesamoid bones within the mandibular adductor muscle system.

## Data Availability

All data generated or analysed during this study are available. The CT scan of specimen FMNH UC 1501 is available on MorphoSource (https://www.morphosource.org/concern/media/000357841), as are the scans of *Manouria impressa* (https://www.morphosource.org/concern/parent/000354073/media/000354076) and *Gopherus polyphemus* (https://www.morphosource.org/concern/parent/000S10605/media/000042629). The CT scan of UMMP 9318 is available in Deep Blue Data, the file hosting service of the University of Michigan (10.7302/74pd-kb09) (CTEES 2022). The 3D models that are newly presented as part of this study are available on MorphoSource (https://www.morphosource.org/projects/000516064).

## Abbreviations

AMNH: American Museum of Natural History, New York City, New York, USA
CM: Carnegie Museum of Natural History, Pittsburg, Pennsylvania, USA
FMNH: Field Museum of Natural History, Chicago, Illinois, USA
NHMUK: Natural History Museum, London, UK
OUMNH: Oxford University Museum of Natural History, University of Oxford, Oxford, United Kingdom
SMF: Naturmuseum Senckenberg, Frankfurt, Germany
UMMP: University of Michigan Museum of Paleontology, Ann Arbor, Michigan, USA
USNM: United States National Museum, Smithsonian Institution National Museum of Natural History, Washington, D.C., USA
